# Steroid hormones and nephrolithiasis: regulation of urine components metabolism and inflammation

**DOI:** 10.1186/s13293-026-00833-9

**Published:** 2026-01-30

**Authors:** Xinrong Zhang, Shuaibin Wang, Jiaxin Zhao, Bingyu Xiang, Mingxia Zhang

**Affiliations:** 1 Shanxi Bethune Hospital, Third Hospital of Shanxi Medical University, Shanxi Academy of Medical Sciences, Tongji Shanxi Hospital, Taiyuan, 030032 China; 2 Academy of Medical Sciences, Shanxi Medical University, Taiyuan, 030001 China; 3 School of Basic Medicine, Shanxi Medical University, Taiyuan, 030001 China; 4 Laboratory Department, Shanxi Provincial Children’s Hospital, Taiyuan, 030001 China

**Keywords:** Nephrolithiasis, Steroid hormones, Oxalate, Phosphate, Calcium, Inflammation

## Abstract

The global incidence of nephrolithiasis has increased significantly in recent decades. The prevalence remains higher in males than females, the exact mechanisms responsible for this gender-based disparity in nephrolithiasis risk remain incompletely understood. Although dietary and lifestyle factors contribute to this difference, they do not entirely account for the observed variation. Emerging evidence suggests that steroid hormones may play a pivotal role in modulating renal stone formation through their influence on calcium, oxalate, and phosphate metabolism, as well as regulating the renal inflammatory microenvironment. This review synthesizes current knowledge on the interplay between steroid hormones and nephrolithiasis pathogenesis, providing a theoretical framework for understanding gender-specific susceptibility and highlighting potential avenues for tailored preventive and therapeutic approaches.

## Highlights

Systematically demonstrating that steroid hormones are key regulators of gender differences in nephrolithiasis fills a research gap where dietary/lifestyle factors alone cannot fully explain these disparities.

Provides a comprehensive framework for the effects of steroid hormones on urine components (oxalate, phosphate, calcium) and inflammation associated with nephrolithiasis.

Provides the target sites and potential mechanisms by which steroid hormones influence nephrolithiasis.

Proposes clinical translation directions for steroid hormones therapy in nephrolithiasis.

It points out the existing controversies regarding the regulatory mechanisms of steroid hormones on nephrolithiasis and offers reasonable speculations on these controversies.

## Introduction

Nephrolithiasis is a urological disease with high global prevalence and significant recurrence rate [1]. Calcium-containing stones, primarily composed of calcium oxalate and calcium phosphate, are the most common type [2, 3]. Stone formation arises not from a single factor, but from the interplay of genetics [4], metabolism [5], environment [6], and diet [7]. Recent studies suggest that nephrolithiasis should be regarded as a systemic disease [8, 9].

Data from the U.S. National Health and Nutrition Examination Survey (NHANES) revealed a significant gender difference in nephrolithiasis incidence (6.5% in females vs. 9.3% in males) between 2008 and 2018 [10, 11]. However, other studies also suggest this gender gap in stone disease is narrowing [12, 13]. In China, a large cross-sectional study further demonstrated that the risk of nephrolithiasis increases steadily with age in females, while in males, the risk rises initially and then declines, which presumably associated with weakened androgen signaling [14]. In addition to potentially influencing social behaviors, such as dietary preferences and occupational exposure [15, 16], while steroid hormones are increasingly recognized to regulate the metabolic homeostasis of lithogenic substances and the local renal microenvironment [17, 18].

This review aims to systematically organize and deeply explore nephrolithiasis and steroid hormones of multidimensional mechanisms. By synthesizing these insights, this review seeks to advance understanding of gender differences in nephrolithiasis and inform the development of gender-specific prevention and treatment strategies.

## Nephrolithiasis and metabolism of urine components: oxalate, phosphate, and calcium

**Fig. 1:**
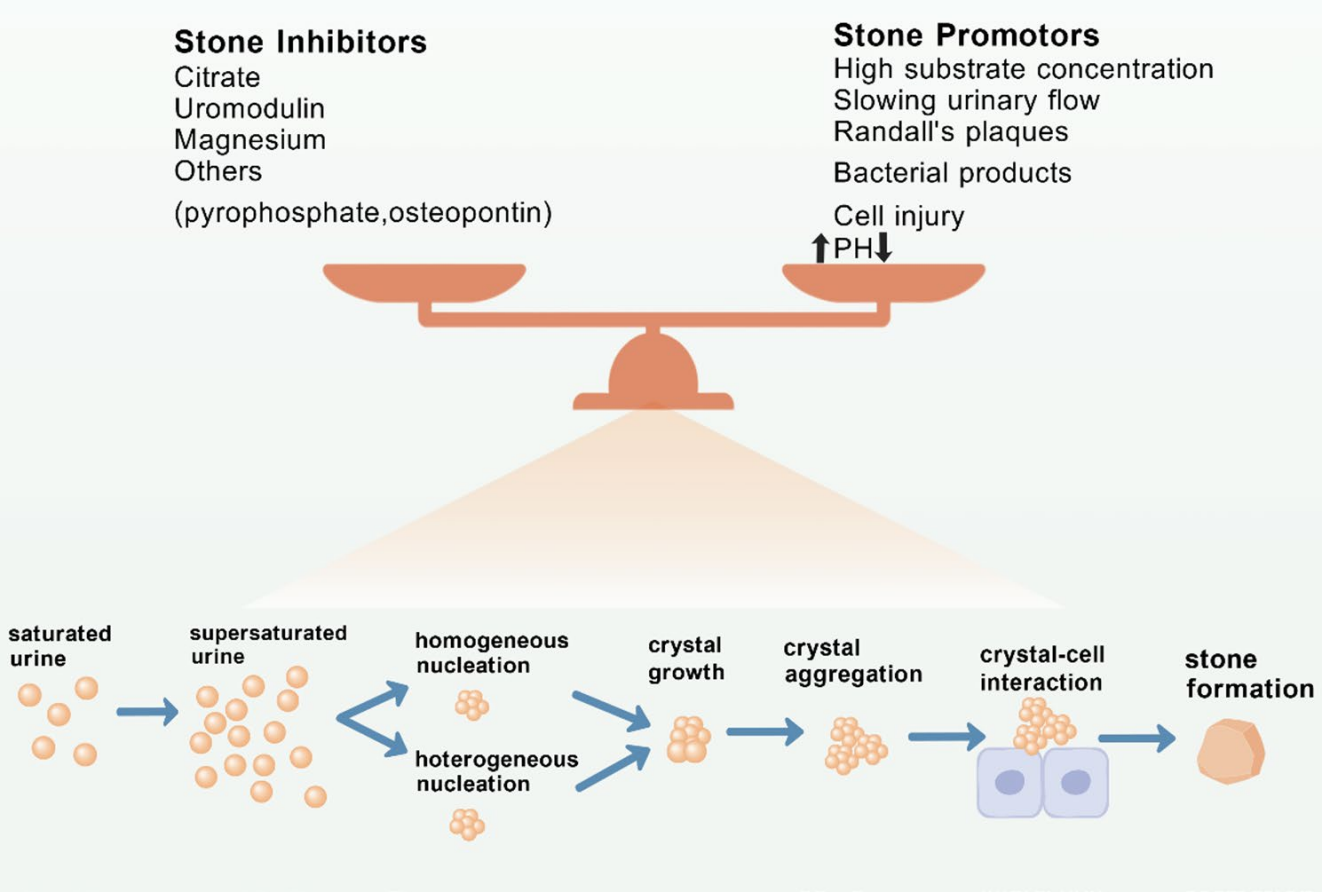
Overview of the pathogenesis of nephrolithiasis

The formation of kidney stones is a complex biologic process (Fig. 1) [19]. Such a process starts with urine supersaturation, followed by crystal nucleation, growth, aggregation, and retention in the kidney [20, 21]. Urinary solute supersaturation provides the necessary milieu for stone formation. So, metabolic imbalances in urine involving oxalate, phosphate, and calcium are crucially important in this context [22].

Oxalate is a core component of calcium oxalate stones, with exogenous sources (such as spinach, amaranth and other foods rich in oxalic acid) and endogenous synthesis (primarily in the liver) [23]. Under physiological circumstances, urinary oxalate concentrations are extremely low. However, metabolic disturbances can trigger a sharp rise in urinary oxalate [24]. This leads to rapid combination with calcium ions, forming highly stable calcium oxalate crystals that facilitate heterogeneous nucleation and growth into the most common type of kidney stones [8, 25].

Phosphate is essential for calcium phosphate stones and Randall’s plaque [8]. In an alkaline urinary environment, the solubility of calcium phosphate decreases, promoting precipitation [26]. Conditions like renal tubular acidosis can create such an environment persistently, fostering Randall’s plaque formation at renal papillae tips [27]. These plaques act as nucleation sites for calcium oxalate crystals, indirectly promoting stone development [8, 28].

Calcium serves as the primary substrate for calcium-containing stones. Under physiological conditions, most filtered calcium is reabsorbed, with minimal urinary excretion [29, 31]. Abnormalities in calcium metabolism, such as excessive intestinal absorption [32], impaired renal reabsorption function [33], or abnormal parathyroid hormone secretion [34], can lead to hypercalciuria. This not only provides core material for stone formation but also reduces the solubility of anions like oxalate, facilitating crystallization [35, 36].

Accordingly, this review will examine the relationship between steroid hormones and nephrolithiasis through the lens of these three key components.

### Steroid hormones and nephrolithiasis: regulation of oxalate metabolism

#### Oxalate metabolism and nephrolithiasis

Transmembrane oxalate transport is mediated by specific transporters, with SLC26A6 and SLC26A1 (Sat-1) being key regulators of intestinal absorption and renal excretion [37]. SLC26A6, a conserved anion transporter (Cl⁻/oxalate) primarily localized to the apical membrane of intestinal epithelial cells and renal proximal tubules, is responsible for intestinal oxalate absorption and renal oxalate secretion [37]. In male SLC26A6 knockout (SLC26A6–/–) mice is demonstrated, which elevated serum and urinary oxalate levels and develop calcium oxalate urolithiasis [38]. Sat-1, a sodium-independent sulfate anion transporter (Oxalate-SO₄²⁻/HCO₃⁻) expressed in the basolateral membrane of renal proximal tubules and the sinusoidal membrane of hepatocytes, is also critical for oxalate homeostasis [39]. Previously, characterization of the Sat1–/– mouse suggested that the loss of Sat1-mediated oxalate secretion by the intestine was responsible for the hyperoxaluria, hyperoxalemia, and calcium oxalate urolithiasis reportedly displayed by this model [40]. However, recent trans-epithelial flux assays using Sat1–/– mice suggest that its regulatory role may be confined to the liver or kidneys. Additionally, Sat1–/– mice were neither hyperoxaluric nor hyperoxalemic. Instead, 24 h urinary oxalate excretion was almost 50% lower than in WT mice [41].

**Fig. 2:**
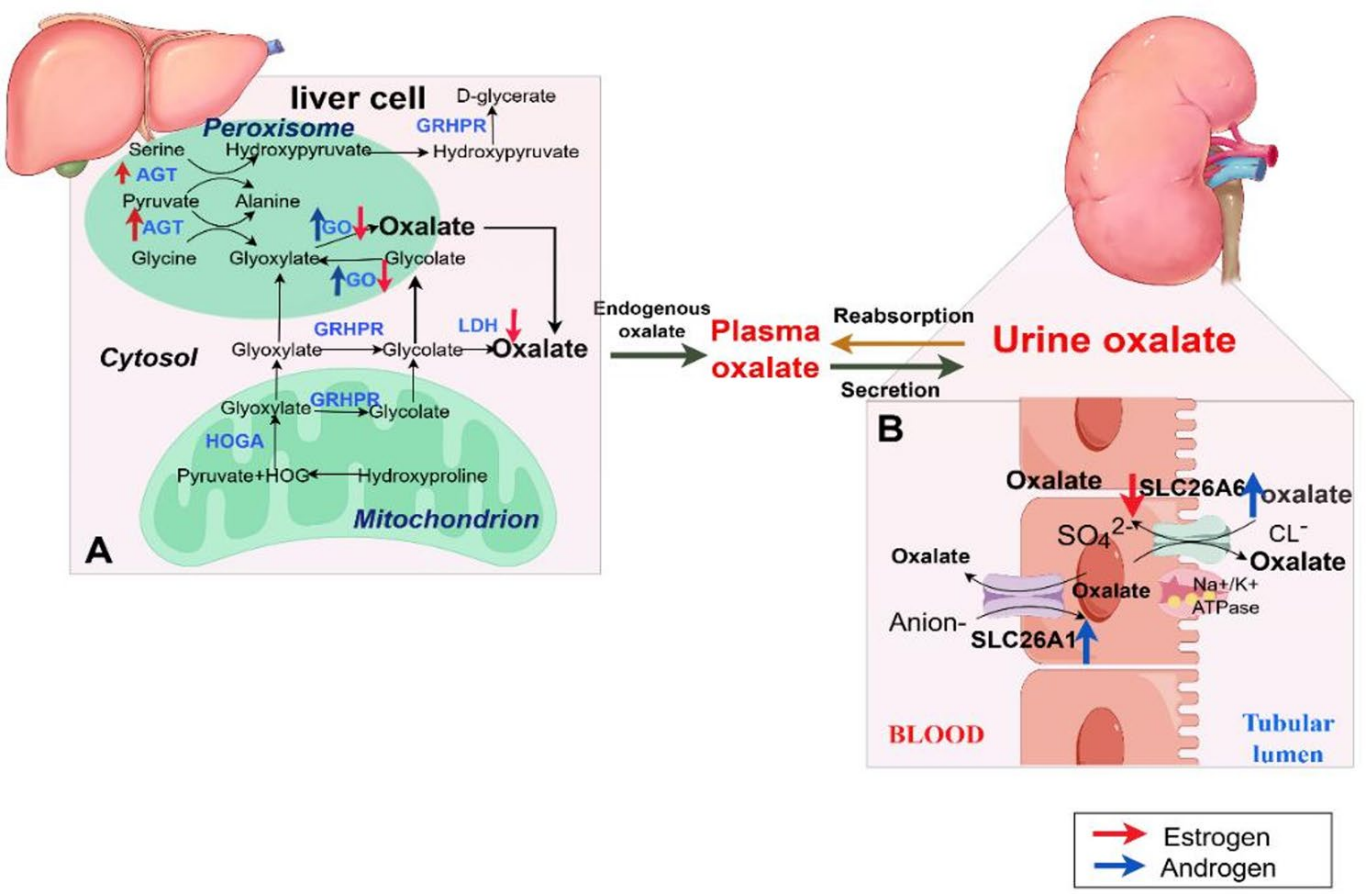
Role of steroid hormones in oxalate metabolism **(A)** Endogenous pathways for oxalate synthesis in hepatocytes. Estrogen (red arrow) promotes AGT while inhibiting GO and LDH, thereby suppressing oxalate synthesis. Androgen (blue arrow) enhances GO, increasing oxalate production. **(B)** Oxalate transport in the renal proximal tubule. Estrogen (red arrow) reduces SLC26A6 transport, decreasing oxalate secretion, while androgen has the opposite effect. Androgen (blue arrow) enhances SLC26A1 transport, increasing blood oxalate concentration. AGT, alanine-glyoxylate aminotransferase; GO, glycolate oxidase; GRHPR, glyoxylate reductase; LDH, lactate dehydrogenase; HOGA, 4-hydroxy-2-oxoglutarate aldolase

The liver is the primary site of endogenous oxalate synthesis, where the balance between oxalate-producing and detoxification pathways directly determines oxalate output (Fig. 2). Primary hyperoxaluria (PH), a rare genetic disorder caused by mutations or defects in liver enzymes, is the primary genetic factor leading to severe calcium oxalate kidney stones and comprises three subtypes [42, 43]. PH1 is characterized by deficiency of alanine-glyoxylate aminotransferase (AGT) [44]. In vivo studies explored the pathway of inhibiting hydroxyfumarate production by targeting hydroxyfumarate oxidase 1 (HAO1) mRNA via Dicer substrate small interfering RNA (DsiRNA), thereby reducing calcium oxalate deposition in PH1 mouse models [45]. HAO1 encodes glycolate oxidase (GO), which catalyzes the oxidation of glycolate into glyoxylate (the direct precursor of oxalic acid). PH2 deficiency involves both carboxymalonic reductase and glyoxylate reductase (GRHPR) [46]. Defective activity in either AGT or GRHPR leads to insufficient removal of glyoxylate, which is then oxidized to oxalate via lactate dehydrogenase (LDH). Increased oxalate synthesis triggers calcium oxalate deposition in kidney [47, 48]. PH3 (mitochondrial 4-hydroxy-2-ketoglutarate aldolase HOGA1 gene mutation) presented clinically with kidney stones disease at age 13 and renal failure at age 33 [49].

#### Role of steroid hormones in oxalate metabolism

A 24-hour urine analysis of 628 kidney stone formers using gas chromatography-mass spectrometry (GC-MS) revealed a positive correlation between urinary oxalate excretion and the excretion of six androgen metabolites [50].

In vivo studies have further elucidated this regulatory relationship. In SLC26A6–/– rats, renal SLC26A6 mRNA and protein expression exhibit male-dominant patterns, and prepubertal rats show low, sex-independent expression, while adult males display high expression [51]. Castration of adult male rats reduces SLC26A6 expression, which is restored by testosterone treatment, indicating that post-pubertal androgens regulate the male-dominant expression of renal SLC26A6 [51]. Conversely, in vitro experiments indicate that estrogen (primarily β-estradiol) reduces SLC26A6 activity in the nephrons, leading to decreased transcellular oxalate secretion and reduced apoptosis rates [52]. Simultaneously, estrogen may also inhibit oxalate absorption by upregulating SLC26A6 expression or function in the intestine [53], or through other intestinal secretion/barrier mechanisms, thereby lowering blood oxalate concentrations. This explains why females typically exhibit smaller increases in urinary oxalate than males following consumption of equivalent high-oxalate diets, reducing oxalate deposition probability [50, 54]. Transport studies in isolated matrix vesicles (which are thought to initiate renal crystal deposition by inducing nucleation and crystallization) confirm that male rats have increased Sat-1 protein expression in the basolateral invaginations of cortical proximal convoluted tubules, contributing to enhanced oxalate biosynthesis [55].

Additional research has underscored the role of steroid hormones in regulating oxalate-synthesizing enzymes. In Sprague-Dawley rats [56], testosterone treatment (in both males and females) significantly increased renal crystal deposition, urinary oxalate excretion, and hepatic/renal GO expression. Conversely, estradiol treatment and male castration reduced these parameters. Another study found that Hao1 mRNA expression is predominantly male-specific and unaffected by ethylene glycol (EG) treatment [57]. Collectively, these data indicate that testosterone promotes renal crystal deposition by upregulating GO expression and enhancing oxalate synthesis, while estradiol acts as an inhibitor. Furthermore, hepatocytes serve as a major site for hepatic enzyme production. In vitro experiments further show that estradiol inhibits LDH production in hepatocytes, protecting hepatocytes against oxidative damage and apoptosis [58].

Liang et al. [59] discovered that androgen receptor (AR) signaling directly upregulates GO in the liver and the NADPH oxidase subunit P22-PHOx in renal epithelium at the transcriptional level, potentially increasing oxalate biosynthesis. In vitro studies [60] indicate that estrogen receptor β (ERβ) reduces hepatic oxalate biosynthesis by increasing AGT expression in hepatocytes, thereby inhibiting oxalate accumulation and preventing nephrolithiasis formation.

In summary, estrogen increases transporter activity and inhibits hepatic oxalate biosynthesis, whereas androgens exert the opposite effect. This can lead to differences in nephrolithiasis risk between males and females (Fig. 2).

### Steroid hormones and randall’s plaques: regulation of phosphate metabolism

#### Phosphate metabolism and randall’s plaque formation

First identified by Alexander Randall in 1937 [61], Randall’s plaques serve as the initiation platform for calcium oxalate stones, forming through three stages: initial calcium phosphate (CaP) crystal formation, progression of interstitial biomineralization, and plaque enlargement with epithelial penetration, ultimately providing attachment sites for calcium oxalate stones development [62].

**Fig. 3:**
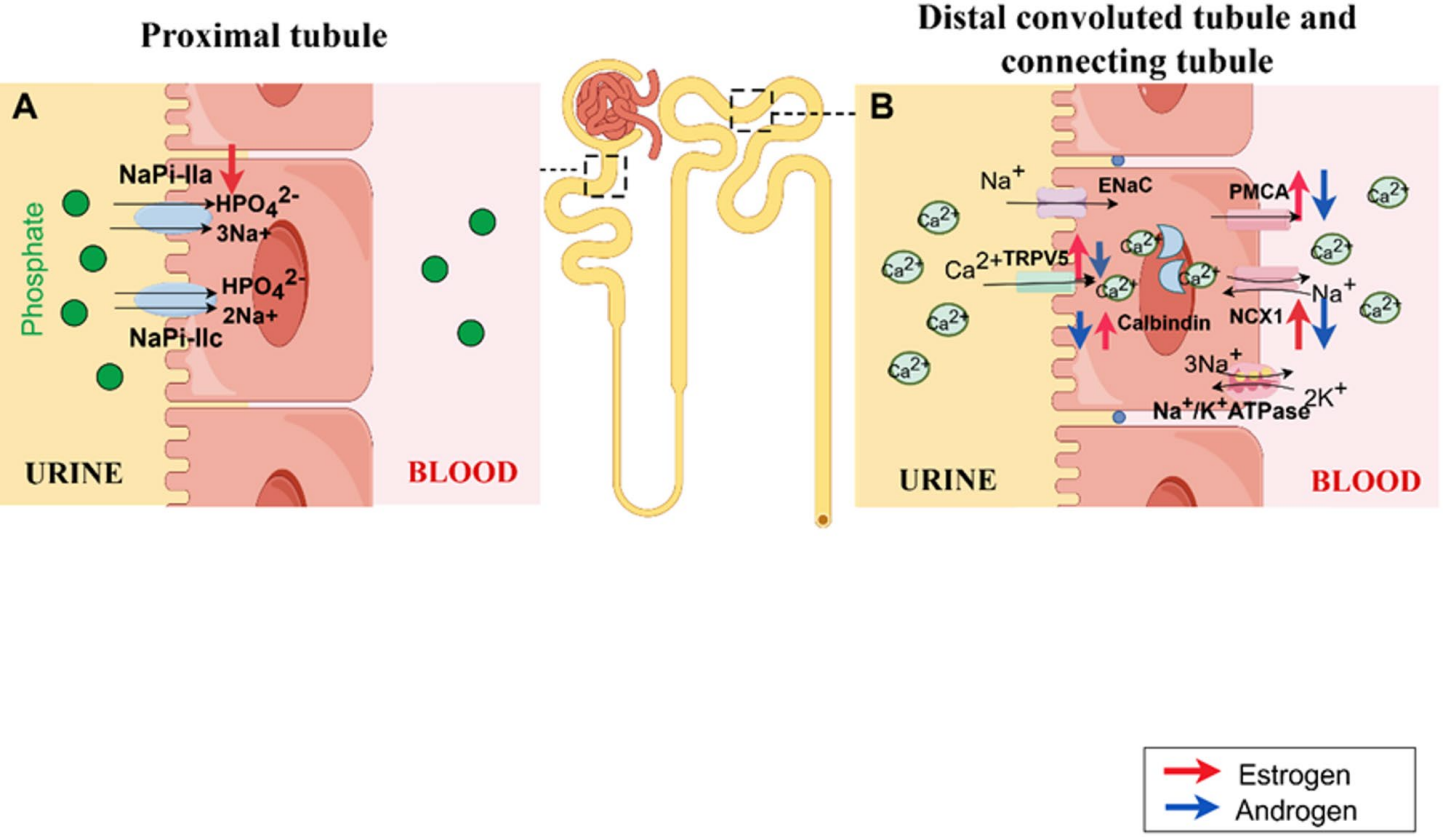
Role of steroid hormones in phosphate and calcium metabolism **(A)** Phosphate transport in the proximal renal tubule. Estrogen (red arrow) reduces NaPi-IIa transport, inhibiting phosphate excretion. (**B)** Calcium transport in the distal convoluted tubule and collecting duct. Estrogen (red arrow) enhances transport via the TPRV5-Calbindin-PMCA/NCX1 pathway, increasing calcium reabsorption and preventing hypercalciuria. Androgen (blue arrow) has the opposite effect, which role in PMCA remains uncertain. NaPi-IIa, sodium- dependent phosphate transport protein IIa; NaPi-IIc, sodium- dependent phosphate transport protein IIc; TRPV5, transient receptor potential vanilloid channel 5; PMCA, plasma membrane calcium-ATPase; NCX1, sodium-calcium exchanger 1

Hyperphosphatemia directly increases the saturation of calcium and phosphate ions in urine, promoting the formation of stones such as apatite and calcitrinite [63]. Phosphate transport in renal and small intestinal epithelial cells is mediated by type II Na-pi co-transporters of the SLC34 family (IIa, IIb, IIc) [64]. Intestinal phosphate absorption is primarily mediated by NaPi-IIb [65], while renal phosphate reabsorption from tubular filtrate is jointly regulated by NaPi-IIa and NaPi-IIc expressed on the brush border membrane of proximal tubule cells [66] (Fig. 3). Although NaPi-IIc contributes to phosphate reabsorption and bone mineralization, clinical data do not clearly link it to nephrolithiasis pathogenesis. Moreover, in NaPi-IIc–/– mice, phosphate homeostasis shows no significant abnormalities, and no stones are found in their kidneys [67]. In contrast, NaPi-IIa–/– mice exhibit hypophosphatemia, increased urinary phosphate excretion, and Randall’s deposits in renal tubules and interstitium [68].

Serum phosphate levels are also regulated by parathyroid hormone (PTH), Klotho (KL), and 1,25-dihydroxyvitamin D₃ [1,25(OH)₂D₃] [69]. 1,25(OH)₂D₃ and low phosphate intake elevate serum phosphate levels by upregulating NaPi-IIb expression, while PTH reduces serum phosphate levels by promoting NaPi-IIa protein degradation and increasing phosphate excretion.

The KL gene plays a critical role in phosphate homeostasis [70]. Although KL is expressed in multiple tissues, its expression levels are highest in the kidneys, parathyroid glands, and choroid plexus. Initially identified as an anti-aging gene, KL deficiency leads to multi-organ dysfunction, including soft tissue calcification and osteomalacia [71]. Recent studies increasingly focus on the association between KL and nephrolithiasis formation [72, 74].

Von Kossa (VK) staining of KL–/– mice reveals extensive renal crystalline deposits, and renal proximal/distal tubules lose differentiation capacity [72], promoting Randall’s plaque formation. Zewu Zhu et al. discovered [74] KL downregulation in Randall’s plaque tissue, negatively correlated with osteogenic markers (MSX2, Runx2, OCN). Human renal interstitial fibroblasts (hRIFs) exhibit stronger osteogenic potential than human proximal tubular epithelial (HK-2) cells, with more pronounced KL of hRIFs decline following osteogenic induction. In co-culture, KL released by HK-2 cells inhibited hRIFs’ osteogenic differentiation by suppressing the Wnt-β-catenin signaling pathway [74]. This establishes KL deficiency as a causative factor in Randall’s plaque formation.

#### Role of steroid hormones in phosphate metabolism

Clinical studies indicate that men undergoing androgen deprivation therapy (e.g., GnRH analog treatment) exhibit elevated serum phosphate levels and enhanced renal phosphate reabsorption [75], while hypogonadal men receiving androgen therapy show a significant reduction in serum phosphate [76]. A randomized controlled trial further found that androgen supplementation in elderly men lowers plasma phosphate levels (without affecting FGF23 or Klotho) [77]. This suggests androgens may promote phosphate excretion by inhibiting renal phosphate reabsorption, potentially alleviating hyperphosphatemia and thereby reducing CaP stone formation, though this remains controversial.

The Rotterdam Study indicated that serum testosterone may partially explain gender differences in serum phosphate, while estradiol may contribute to gender differences in serum calcium [78]. Postmenopausal women experience a sudden decline in estrogen, leading to elevated serum calcium, phosphate concentrations, and calcium-phosphate product [79], thereby increasing the risk of nephrolithiasis. Women receiving estrogen therapy exhibit reduced serum phosphate levels, as estrogen inhibits NaPi co-transporter in renal proximal tubules [80]. In vivo and vitro studies confirm that rat proximal tubule (PT) cells express both ERα and ERβ, and 17β-estradiol (17β-E_2_) induces phosphaturia by directly and specifically targeting NaPi-IIa in PT cells [81, 82]. Estrogen also causes phosphaturia and hypophosphatemia in mice by downregulating NaPi-IIa and NaPi-IIc proteins in proximal tubules via ERα activation. And, this is independent of PTH levels [83].

Serum phosphate levels decrease with age in both genders [84]. Estrogen reduces phosphate excretion by downregulating NaPi-IIa (studies on androgen effects on NaPi-IIa are relatively scarce), NaPi-IIa downregulation is identified as a risk factor in renal calculi research. Notably, both human and animal studies indicate that due to higher urinary pH in females [85], young females (with normal estrogen secretion) are more prone to CaP crystal formation, while males are more susceptible to CaOx crystals [86, 87]. Disrupted phosphate levels clearly contribute to nephrolithiasis formation or renal tissue damage. Nevertheless, the relationship between steroid hormones, phosphate homeostasis, and Randall’s plaque formation requires more comprehensive research to validate.

A cross-sectional study reported that a significant association between KL and steroid hormones in the U.S. male population. KL levels increased with total testosterone, estradiol and sex hormone-binding globulin levels [88]. However, male testosterone supplementation does not affect KL levels [77]. In response to mild phosphate challenges, females (especially aged females) maintain normal serum phosphorus through significant upregulation of FGF23, forming a protective mechanism of compensatory FGF23 elevation [89]. Males, conversely, rely on maintaining KL expression to ensure FGF23 signaling efficiency, yet their serum FGF23 levels are markedly lower than females (approximately 50% in aged males with high phosphorus compared to aged females with high phosphorus) [89]. This disparity suggests that despite higher KL levels in males, insufficient FGF23 substrate may compromise the long-term stability of FGF23-KL signaling. In contrast, females’ compensatory elevation of FGF23 enables a more proactive response to phosphorus load, reducing renal phosphorus reabsorption pressure and indirectly lowering the risk of renal injury [90]. Therefore, variations in KL expression may contribute to the greater severity of renal injury in males compared to females during progressive kidney disease [91]. Based on this, it can be inferred that estrogen may maintain serum Phosphate levels within the normal range via KL, thereby preventing mineral metabolism disorders.

Notably, in the proximal tubule epithelial cells of KL–/– mice, NaPi-IIa cotransporter expression is elevated, leading to hyperphosphatemia, which an effect reversed by dual knockout of NaPi-IIa and KL mice. Double knockout mice also exhibit reversed aging characteristics, suppressed renal calcification, and significantly extended lifespan, but high-phosphate diets restore premature aging phenotypes [92]. This confirms that phosphate toxicity is a primary cause of premature aging, inducing renal tubular epithelial cell death and other cytotoxic effects that disrupt mineral metabolism and exacerbate chronic kidney disease [93, 94].

In summary, for nephrolithiasis patients, potential mechanisms of estrogen therapy may include: (i) downregulating NaPi-IIa expression to inhibit phosphate toxicity from hyperphosphatemia [82], (ii) upregulating KL expression to prevent cellular/tissue senescence and death, and inhibiting osteogenic differentiation of hRIFs [74]. While this strategy has not been applied to nephrolithiasis treatment, similar mechanisms have been validated in estrogen therapy for acute heart failure [95]. Their experiment demonstrated that 17β-estradiol treatment suppressed adrenal NaPi-IIa expression, normalizing serum phosphorus levels and reversing cardiac mitochondrial respiratory enzyme dysfunction, excessive ROS production, oxidative stress, and cardiomyocyte apoptosis in KL–/– mice. Given the strong association between nephrolithiasis and cardiovascular disease [96, 97], estrogen’s potential role in treating nephrolithiasis via this mechanism merits further in-depth investigation (Fig. 3).

### Steroid hormones and calcium-containing stones: regulation of calcium metabolism

#### Calcium metabolism and nephrolithiasis formation

Renal calcium reabsorption via two pathways, with dysfunction in either disrupting calcium balance and increasing stones risk [98]. Paracellular passive transport primarily occurs in the proximal tubule (PT, 60–70%) and the thick ascending limb of the loop of Henle (TAL, 20%) [99].

Transcellular active transport occurs primarily in the distal convoluted tubule (DT, 10%) and collecting duct (CT, 5%) [100] (Fig. 3). Calcium influx across the apical membrane of DT cells is mediated by the transient receptor potential vanilloid channel 5 (TRPV5), followed by intracellular transport via Calbindin-D28k (in DT principal cells) or Calbindin-D9k (in connecting tubules). Finally, calcium is transported into the bloodstream via plasma membrane calcium-ATPase (PMCA) and sodium-calcium exchanger 1 (NCX1) in the basolateral membrane [101].

Dysfunction of the TRPV5-Calbindin-PMCA1b transport pathway is directly implicated in idiopathic hypercalciuria kidney stones disease [102, 103]. TRPV5–/– mice confirm that TRPV5 is a key regulator of renal tubular Ca²⁺ reabsorption [104], altered TRPV5 expression disrupts pathway integrity, leading to tubular calcium deposition and stone formation. Abnormal TRPV5 expression or function is also directly associated with increased recurrence risk in calcium-containing nephrolithiasis patients [105].

1,25(OH)₂D₃ and PTH also participate in calcium homeostasis regulation [36, 106]. 1,25(OH)₂D₃ is a key mediator of vitamin D’s effects on calcium and bone metabolism [107]. In clinical trials, multivariate regression analysis demonstrated a positive correlation between 1,25(OH)₂D₃ and urinary calcium excretion [108]. Shakhssalim et al. identified 1,25(OH)₂D₃ as a key hormone in recurrent nephrolithiasis pathogenesis, increasing stone risk by increasing urinary calcium excretion [109]. In both humans and rats, vitamin D supplementation has been shown to cause hypercalciuria, nephrolithiasis, and/or nephrocalcinosis [110, 111]. Vitamin D deficiency (defined as 25(OH)D < 20 ng/mL) is significantly more prevalent in nephrolithiasis patients than in non-stone patients, and may elevate PTH levels, further increasing stone risk [112, 113]. PTH converts 25-hydroxyvitamin D[25(OH)D] to active 1,25(OH)₂D₃ to promote intestinal and renal calcium absorption, and stimulates bone resorption to raise serum calcium [114]. Overactivation of PTH’s calcium-raising effects leads to accelerated bone resorption (elevating serum calcium) and inhibited renal calcium reabsorption (increasing urinary calcium), promoting crystal formation with oxalate and phosphate, and ultimately stone development [108, 115, 116]. Clinically, patients with primary hyperparathyroidism (excessive PTH secretion) exhibit a significantly increased incidence of nephrolithiasis [34, 117, 119].

#### Role of steroid hormones in calcium metabolism

Steroid hormones (estrogens, androgens) regulate renal calcium transport through direct or indirect pathways (Fig. 3), though results remain controversial due to experimental heterogeneity (e.g., calcium loading, species, model types), making calcium metabolism a key focus of current research.

Mouse models lacking aromatase (with impaired estrogen synthesis) exhibit hypercalciuria and significantly reduced expression of renal transporters TRPV5, PMCA1b, NCX1, and Calbindin-D28k. Treatment with E_2_ fully restored the function of transporters including TRPV5 [120]. Following ovariectomy in mature rats, estradiol (E_2_) supplementation at varying doses increases serum calcium, reduces urinary calcium excretion, and upregulates renal transporters including TRPV5, with no significant changes in protein levels following 1,25(OH)₂-vitamin D₃ addition, indicating this process is independent of the vitamin D pathway [121]. These findings demonstrate that estrogen directly stabilizes Ca²⁺ homeostasis by regulating calcium transporters in DT and CT, inhibiting nephrolithiasis development.

Estrogen-deficient female rats fed low-calcium diets exhibit negative calcium balance, accompanied by downregulation of TRPV5, CaBP28k, and PMCA1b, increasing stone risk [122]. In contrast, ovariectomized rats on high-calcium diets show positive calcium balance, suggesting that estrogen deficient-mediated regulation of calcium metabolism may be influenced by calcium intake levels [122].

A randomized controlled trial of 36,282 postmenopausal women found that the intervention group (500 mg calcium carbonate + 200 IU vitamin D₃ twice daily, achieving an average daily calcium intake of 2100 mg) had a 17% higher risk of nephrolithiasis than the placebo group after 7 years of follow-up [123]. An observational analysis from the Nurses’ Health Study I (NHS I) reached similar conclusions: women taking calcium supplements had a 20% increased risk of stone events [124]. However, data from the Health Professionals Follow-up Study (45,619 healthy men without stone history) showed that the low-calcium diet group (797 ± 85 mg/day) had a 50% lower stone risk than the normal intake group (1307 ± 280 mg/day) [125]. Another randomized controlled trial in patients with recurrent calcium oxalate stones and hypercalciuria confirmed that the balanced calcium group (1200 mg/day) exhibited significantly reduced urinary oxalate excretion and a 50% lower risk of new stones after 5 years, while the ultra-low calcium group (400 mg/day) showed higher urinary oxalate excretion [126]. Collectively, balanced calcium intake has become a core component of dietary recommendations for nephrolithiasis patients, as it reduces stone formation risk by maintaining calcium metabolic balance.

Postmenopausal women face increased risks of fractures and osteoporosis, primarily due to uncontrolled osteoclast activity (bone resorption exceeding formation), reduced vitamin D activation, and diminished calcium reabsorption efficiency, leading to rapid bone loss, hypercalciuria, and elevated nephrolithiasis risk [127, 128]. The NHS I confirmed that both natural and surgical menopause are associated with higher nephrolithiasis incidence [129]. In vitamin-deficient mice, reduced testosterone and estrogen levels have also been observed [130]. However, the Women’s Health Initiative (WHI) clinical trial found that vitamin D and calcium supplementation inhibited fractures but increased nephrolithiasis risk [123]. In women receiving estrogen therapy, elevated 1,25(OH)₂D₃ levels reduced urinary calcium excretion, decreasing hypercalciuria incidence [131]. A network meta-analysis exploring the efficacy of drug therapies in preventing fractures in postmenopausal women noted that estrogen reduces fracture incidence [132].Therefor, maybe prioritizing estrogen for treating osteoporosis or nephrolithiasis in postmenopausal women, as it elevates 1,25(OH)₂D₃ levels without the side effects of calcium/vitamin D supplementation. However, estrogen dosage must be carefully managed. Tissue-selective estrogen complexes (TSECs) [133], combining selective estrogen receptor modulators (SERMs) with one or more estrogens, have been used for fracture/osteoporosis treatment, offering better tolerance and safety than monotherapy by exerting neutral or antagonistic effects on tissues where estrogen action is undesirable (breast and endometrium) [134].

While urinary calcium excretion is consistently higher in males than females, men with low testosterone levels exhibit a lower risk of nephrolithiasis [50]. Immunohistochemical studies have linked nephrolithiasis to upregulated AR expression in renal tissue [135], and AR–/– mice show significantly reduced calcium oxalate crystal formation [59]. A cross-sectional study further demonstrated a positive correlation between serum testosterone levels and urinary calcium excretion in males, suggesting androgens may directly inhibit renal Ca²⁺ reabsorption via AR, increasing urinary Ca²⁺ saturation and stone risk [136].

In vitro experiments showed that dihydrotestosterone (DHT) treatment of rabbit DCT-CT cells inhibited apical-to-basolateral calcium transport (PMCA) [137]. In contrast, immortalized DT cells incubated with T and DHT for 24 h exhibited increased PMCA activity, enhancing calcium uptake [138]. While T is the primary circulating androgen in male, exerting effects via the AR by converting (through 5α-reductase) to DHT or via ER (following aromatization to estradiol) [139]. DHT possesses 2–3 times the biological activity of T and exhibits higher affinity for target tissues [140]. Additionally, animal studies suggest finasteride (a 5α-reductase inhibitor) may be used to treat nephrolithiasis [141]. Animal studies have yielded conflicting results. Hypercalciuria induced in male rats 2 [142] and 8 weeks [143] after orchidectomy was reversed by treatment with either T or DHT. another study found that castration in male mice reduced urinary calcium excretion and increased expression of transporters like TRPV5, while T supplementation increased urinary calcium excretion and downregulated these transporters [137]. Rougin Khalil et al. found that castrated rats develop hypercalciuria, which is prevented by bisphosphonates (inhibitors of bone resorption). Moreover, combining bisphosphonate treatment with an extremely low-calcium diet did not reduce urinary calcium levels post-castration. This suggests androgens may interact dynamically with renal, skeletal, and intestinal calcium homeostasis [144].

Androgens may indirectly regulate calcium transport by influencing vitamin D metabolism. Therefore, in the presence of androgens in PT, the synthesis of 1,25(OH)₂D₃ is inhibited. This leads to impaired active phosphate reabsorption at the proximal tubule level while simultaneously suppressing active calcium reabsorption at DT level, triggering hypercalciuria and other metabolic abnormalities [144]. In pregnancy, hyperandrogenism may adversely affect placental vitamin D metabolism. Studies reveal testosterone significantly inhibits CYP27B1 (which synthetic vitamin D) while stimulating CYP24A1 (which degrades vitamin D) expression in cultured trophoblasts [145].

In summary, estrogen tends to reduce urinary calcium by enhancing renal calcium reabsorption, decreasing bone calcium loss, and upregulating calcium transporters. However, the effects of androgens on calcium metabolism remain contradictory. First, calcium homeostasis results from the coordinated actions of the kidneys, bones, and intestines. Second, within tissues, T can act as either T or DHT via the AR, or as estradiol via the ER. Further rigorous research is needed to clarify the precise role of steroid hormones in calcium metabolism and nephrolithiasis formation.

## Steroid hormones and nephrolithiasis: regulation of inflammation

**Fig. 4:**
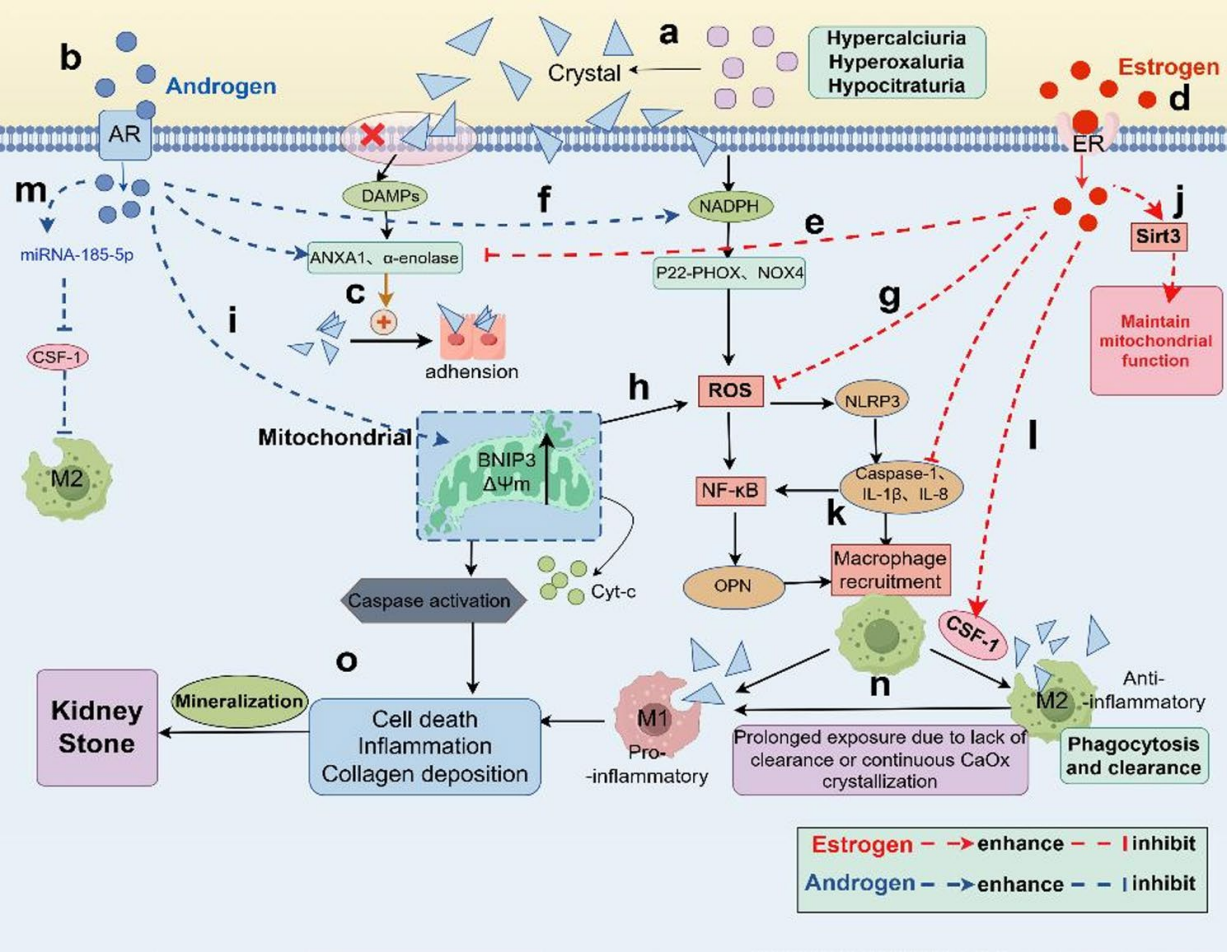
Role of steroid hormones in Inflammation **(a)** Hyperoxaluria, hypercalciuria and hypocitraturia are risk factors involved in the pathogenesis of nephrolithiasis and can lead to epithelial cell damage. Androgens (Blue, **b**) bind to the androgen receptor (AR), increasing α-enolase and promoting adhesion between crystals and cells **(c)**. Estrogens (Red, **d**) bind to the estrogen receptor (ER), inhibiting adhesion between crystals and cells **(e)**. NADPH is a key contributor to ROS-induced inflammation, promoted by androgens **(f).** Estrogen inhibits the production of ROS **(g)**. Mitochondria are also major ROS sources **(h)**. Androgen impair mitochondrial function by increasing BNIP3 and ΔΨm, elevating ROS and accelerating apoptosis **(i)**. Conversely, estrogens enhance Sirt3 to maintain mitochondrial function **(j)**. Inflammatory factors produced during inflammation recruit macrophages **(k)**. Estrogen promotes anti-inflammatory M2 macrophages to phagocytose crystals via CSF-1 **(l)**, while androgens suppress M2 by increasing miRNA-185-5p expression, which reduces CSF-1 expression and increases pro-inflammatory M1 macrophages **(m)**. The increased pro-inflammatory macrophages further sustain the inflammatory microenvironment, promoting crystal nucleation and adhesion in a self-perpetuating cycle**(n)**. Pro-inflammatory macrophages, mitochondrial damage, and the accompanying inflammation drive collagen deposition and mineralization **(o)**, ultimately leading to nephrolithiasis formation. ΔΨm, mitochondrial membrane potential; CSF-1, colony-stimulating factor 1

Nephrolithiasis formation and inflammatory responses exist in a vicious cycle of bidirectional regulation [146]. After calcium oxalate and calcium phosphate crystals precipitate, they damage renal tubular epithelial cells and activate inflammatory pathways, while inflammation further exacerbates crystal deposition and tissue injury [8, 147, 148]. steroid hormones exert effects on this inflammatory process through receptor-mediated pathway regulation, with estrogen exhibiting anti-inflammatory properties and androgens promoting inflammation, thereby further influencing stone progression (Fig. 4).

### Inflammation and stones formation and growth

When calcium oxalate, calcium phosphate, and other crystals become supersaturated and precipitate in urine, the positive charge on their surface interacts with the negative charge of phospholipids on renal tubular epithelial cell membranes, disrupting membrane integrity and releasing damage-associated molecular patterns (DAMPs) such as ATP and HMGB1 [149]. This interaction also exposes annexin A1 [150] and α-enolase [151], enhancing crystal-cell adhesion. Additionally, crystals activate NADPH oxidase within tubular epithelial cells (e.g., p22-phox, NOX4 subunits), promoting reactive oxygen species (ROS) production [152, 153].

In nephrolithiasis pathophysiology, NADPH oxidase is the primary renal source of ROS, while mitochondrial ROS (mtROS) is a significant secondary contributor to ROS-related renal injury [153]. ROS not only directly oxidatively damage cell membranes but also activates the TXNIP-NLRP3 pathway, generating mature pro-inflammatory factors (such as IL-1β, IL-8, and IL-10) that are secreted extracellularly, recruiting immune cells like monocytes and neutrophils to infiltrate the renal interstitium [154, 155]. Furthermore, impaired clearance of damaged mitochondria (due to autophagy dysfunction) releases mtDNA and cytochrome C (initiating apoptosis), exacerbating cell detachment and dysfunction [156, 157]. Crystal stimulation or factors like IL-1β can activate NF-κB inflammatory pathways, upregulating expression of pro-inflammatory genes including TNF-α, IL-6, and MCP-1 (monocyte chemotactic protein-1), further amplifying inflammatory infiltration [158, 159].

### Role of steroid hormones in inflammation

Estrogens exert their effects primarily through two nuclear receptors (ERα, ERβ) and a membrane receptor (GPER) [160]. ERβ is highly expressed in renal tissues (renal tubular epithelial cells, macrophages) and serves as a key anti-inflammatory receptor [161, 162]. Upon binding to ERβ, estrogens directly regulate the transcription of anti-inflammatory genes (such as IL-10, TGF-β) or suppress pro-inflammatory gene expression by interacting with other transcription factors (e.g., AP-1) [163, 164]. Estrogen also protects against acute kidney injury via ERα, though ERα has weaker direct pathological relevance to stone formation than ERβ [165]. In hyperoxaluria-induced ERβ−/−mice, renal IL-1β and TNF-α levels are significantly increased, with calcium oxalate deposition reaching 2–3 times higher than in wild-type mice. While ERα−/− mice exhibit only mildly abnormal inflammatory markers, which could be improved through ERβ compensation [60]. Thus, ERβ plays a dominant anti-inflammatory role in the kidney, while ERα is secondary. GPER also participates in inflammatory responses. The GPER1 agonist G-1 protect human renal tubular epithelial cells from damage [166].

Estrogen reduces renal inflammatory damage by upregulating antioxidant enzyme activity (SOD1, CAT) in renal tissue, decreasing ROS production [167, 168]. In vitro studies revealed [169] that E_2_ treatment reduced the release of annexin A1 and α-enolase from renal tubular epithelial cells under hyperoxalic acid conditions, decreases TLR4/NF-κB activity, and ultimately diminishes IL-1β and IL-18 release, inhibiting crystal adhesion to tubular epithelial cells and enhancing cell proliferation and tissue healing [151]. Postmenopausal women exhibit a twofold increase in nephrolithiasis incidence, accompanied by markedly elevated urinary inflammatory markers [50].

In contrast, androgens are key factors inducing renal injury. AR are widely distributed in renal tubular epithelial cells, renal interstitial macrophages, and hepatocytes [170]. Studies in AR−/− rat models confirm that AR is associated with upregulation of nephritis markers, cell death, and fibrosis markers [171]. Liang et al. discovered that androgen signaling pathways directly upregulate hepatic GO and the renal epithelial NADPH subunit P22-PHOx at the transcriptional level, potentially enhancing oxalate biosynthesis and leading to nephrolithiasis [59]. Androgens also increase surface α-enolase expression, enhancing calcium oxalate crystal adhesion to the apical membrane of renal tubular epithelial cells [172]. Another newly developed androgen receptor degradation enhancer, dimethylcurcumin (ASC-J9), has been shown to inhibit oxalate crystal formation by modulating oxalate biosynthesis and ROS-induced injury in rat renal tubular epithelial cells [173, 174]. Proteomics analysis of individuals receiving sex steroid hormone therapy (estradiol, testosterone) revealed that proteins involved in endothelial function (SFRP4, SOD3), anti-inflammation (TSG-6), and renal tissue structure maintenance are positively correlated with estradiol but negatively correlated with testosterone. Additionally, testosterone elevates levels of tubular injury biomarkers (YKL-40, MCP-1), further confirming estrogen’s protective role for tubular epithelial cells, while androgens exert the opposite effect [175].

Mitochondria, cellular powerhouses and major ROS sources, play a pivotal role in initiating kidney stone-related inflammation, crystal adhesion, and cellular damage-induced apoptosis [176]. Estrogen exerts mitochondrial protective effects via ER by upregulating mitochondrial autophagy-related proteins to promote clearance of damaged mitochondria and enhancing mitochondrial antioxidant capacity [177]. In contrast, androgens exacerbate mitochondrial dysfunction via AR. SIRT3, a nicotinamide adenine dinucleotide (NAD)-dependent class III histone deacetylase, functions within mitochondria to enhance respiration and reduce ROS production [178]. In acute kidney injury models, sex steroid hormone-dependent differences in renal mitochondrial Sirt3 (mtSirt3) expression have been observed that estradiol increases mtSirt3 protein and testosterone decreases it [179]. BNIP3, a gene mediating necrotic-like cell death by inducing mitochondrial permeability transition pore opening and mitochondrial dysfunction [180, 181]. In human and mouse renal tubular epithelial cell (RTEC) assays, mitochondrial membrane potential (ΔΨm) was significantly higher in the testosterone-treated group than in the normal control group, while BNIP3 siRNA treatment reduced ΔΨm compared to control and negative control siRNA groups [182]. Thus, testosterone induces apoptosis via BNIP3-mediated mitochondrial dysfunction, and RTEC death represents a key pathophysiological process in renal stone development [158]. And, testosterone promotes apoptotic damage in human renal tubular cells [183]. Sex steroid hormone-mediated differential regulation of mitochondria and related genes may serve as potential therapeutic targets to mitigate excessive ROS-induced renal injury and apoptotic shedding.

As previously described, disproportionate calcium oxalate exposure activates the NF-κB pathway, triggering the production of numerous inflammatory cytokines [184] that damage mitochondria (generating ROS) and induce macrophage recruitment, depending on cytokine concentration, exposure duration, and cytokine competition [154]. M1 macrophages promote renal crystal development, while M2 macrophages reduce pro-inflammatory factor expression through crystal phagocytosis and inhibit stone formation [185, 186].

Studies using mouse macrophages (RAW264.7 and female murine bone marrow macrophages) show that 24-hour E_2_ treatment upregulates Sirt3 and attenuates oxidative stress and pro-inflammatory polarization in M1 macrophages [187]. Estrogen also upregulates CSF-1 (colony-stimulating factor 1) receptor expression on macrophage surfaces, enhancing CSF-1-mediated M2 polarization signaling [188]. M2 induce secretion of anti-inflammatory factors such as IL-10 and TGF-β, and enhancing macrophage phagocytic capacity toward calcium oxalate crystals [189]. Androgens promote M1 macrophage activation via AR while inhibiting M2 polarization, disrupting the renal inflammatory microenvironment balance and suppressing intrarenal macrophage phagocytosis of CaOx crystals [188]. Mechanistic analysis indicates that AR reduces macrophage CSF-1 expression via increased miRNA-185-5p expression, inhibiting M2 macrophage polarization-mediated phagocytosis of CaOx crystals [174]. A prospective study further found that CaOx renal stones are positively correlated with genetic expression of AR and miRNA, and inversely related to CSF-1 [190]. Collectively, immunoregulation of inflammatory cytokines offers novel insights into macrophage-mediated prevention and treatment of nephrolithiasis, with inflammatory cytokines potentially representing a key pathway through which steroid hormones influence macrophage polarization in nephrolithiasis disease.

Existing research indicates a close relationship between vascular calcification and nephrolithiasis, with vascular calcification accelerating the stone progression, while nephrolithiasis also often accompanied by vascular calcification [190, 192]. Osteopontin (OPN) and matrix Gla protein (MGP) are two crucial calcification inhibitors in the human body, preventing both vascular calcification and nephrolithiasis [193, 194]. Animal studies indicate that compared to mice with normal OPN expression, high-phosphate-fed OPN−/− mice exhibit more severe vascular calcification and renal calcification, suggesting OPN can simultaneously prevent nephrocalcinosis and vascular calcification [195]. Studies on Sprague-Dawley rats before and after castration indicate that estrogen inhibit stone formation by increasing renal OPN expression and reducing urinary oxalate excretion, while androgens exert the opposite effect [18]. Miyajima et al. also demonstrated in a hyperoxaluric rat model that exogenous estrogen administration to the ovariectomized (OVX) rat increased OPN mRNA expression, consistent with results from primary cultured rat renal cells. Furthermore, ERα protected renal epithelial cells from damage in the presence of estrogen [196]. In vascular calcification, estrogen maintains vascular smooth muscle cell (VSMC) functional integrity by promoting OPN expression via G protein-coupled receptor 30 (GPR30) [197]. MGP is the most potent inhibitor of tissue calcification [194, 198]. MGP-deficient mice spontaneously develop arterial calcification [199]. Numerous studies have linked MGP gene polymorphisms to increased nephrolithiasis susceptibility [200, 201]. Estrogen also increase MGP expression, slowing phosphate-induced osteogenic differentiation of VSMCs [202], delaying the progression of nephrolithiasis.

In summary, estrogen suppresses nephrolithiasis-related inflammation through ER anti-inflammatory pathways, mitochondrial protection, and M2 macrophage polarization, whereas androgens exert the opposite effect (Fig. 4).

## Clinical prospects and challenges of sex hormone therapy for nephrolithiasis

A clinical cross-sectional study employing a questionnaire survey finds that natural and surgical menopause are associated with nephrolithiasis, while postmenopausal estrogen therapy showed no association with stone risk in postmenopausal women [131]. Similarly, in 91,731 female Nurses’ Health Study participants, estrogen use was unrelated to nephrolithiasis occurrence in postmenopausal women [203]. However, retrospective studies suggest that estrogen therapy in postmenopausal women with nephrolithiasis may reduce recurrence risk by lowering urinary calcium and calcium oxalate saturation [204]. Despite this potential benefit, estrogen replacement therapy is limited as a routine preventive measure for nephrolithiasis due to increased risks of thrombosis and breast cancer [205]. The combined use of estrogen and selective estrogen receptor modulators (SERMs) may offer a promising alternative for treating nephrolithiasis while avoiding some side effects, though this approach has not been explored in nephrolithiasis-related studies [133, 206, 207]. It has, however, been applied to the treatment of other related kidney diseases (such as renal ischemia-reperfusion injury and chronic kidney disease) [165, 208, 209]. Developing strategies that provide renal protection while avoiding systemic side effects represents an important future research direction.

Given androgens’ active role in promoting stone-forming substance excretion and renal inflammation, antagonizing the AR signaling pathway theoretically serves as a strategy for preventing and treating male nephrolithiasis. However, systemic AR antagonists (such as bicalutamide) are associated with side effects such as decreased libido and gynecomastia, limiting their use exclusively for stone prevention [210, 212]. Finasteride (a 5α-reductase inhibitor) inhibits testosterone-induced calcium oxalate crystallization and crystal-cell adhesion [141]. Novel AR degradation promoters like ASC-J9 show promising efficacy in kidney disease [59, 173]. ASC-J9 suppresses renal cell carcinoma progression by targeting an AR-dependent HIF-α/VEGF signaling pathway [213] and may reduce intrarenal CaOx crystal deposition by altering macrophage recruitment/M2 polarization [174]. However, its safety and efficacy require validation through human clinical trials.

Phytoestrogens, natural plant-derived compounds with a structure resembling estradiol, can bind to ER and exert estrogenic effects [214, 216]. Daidzein, a soy isoflavones, exerts pharmacological effects by binding to ERα and β [217]. In male menopausal rat models, daidzein upregulates the anti-aging protein KL and the NaPi-IIa cotransporter, stabilizing calcium and phosphate homeostasis. Notably, unlike estrogen therapy (which reduces NaPi-IIa levels), daidzein may exert rapid non-genomic effects by activating G-protein-coupled receptor 30 (GPR30/GPER) [218], while estradiol treatment activates the mitogen-activated protein kinase (MEK 1/2) [219]. Trigonelline, the second most abundant bioactive alkaloid in coffee, is also classified as a phytoestrogen. By inhibiting the formation, growth, and adhesion of calcium oxalate crystals to cells, while simultaneously downregulating crystal receptor expression, the formation of nephrolithiasis is suppressed [220]. Kaempferol, one of the most common flavonoids, inhibits AR expression, oxidative stress and inflammation by regulating the AR/NOX2 signaling pathway, reducing CaOx crystal deposition and crystal-induced kidney damage, and inhibiting stone formation [217].

In summary, based on the established core regulatory role of steroid hormones in nephrolithiasis formation, targeting sex hormone signaling pathways may emerge as a potential new strategy for prevention and treatment.

## Conclusion

The prevalence of nephrolithiasis in women has risen significantly, narrowing the gender gap. Women today bear a heavier burden regarding obesity, weight-loss surgery, and dieting. Compared to men, women have higher urinary pH levels, leading to greater absorption of dietary organic anions, all factors associated with increased stone formation. However, research indicates that several differences in stone formation mechanisms persist between men and women. Increasing evidence confirms the influence of steroid hormones on stone formation and the associated inflammatory microenvironment. Furthermore, these gender differences in stones may offer new insights for developing personalized treatment strategies in the future.

## Data Availability

No datasets were generated or analysed during the current study.

## Abbreviations

1,25(OH)₂D₃: 1,25-dihydroxyvitamin D₃
25(OH)D: 25-hydroxyvitamin D
AGT: Alanine-glyoxylate aminotransferase
AR: Androgen receptor
CaP: Calcium phosphate
CKD: Chronic kidney disease
CSF-1: Colony-stimulating factor 1
CT: Collecting duct
DAMPs: Damage-associated molecular patterns
DsiRNA: Dicer substrate small interfering RNA
DT: Distal convoluted tubule
ΔΨm: Mitochondrial membrane potential
17β- E_2_: 17β-estradiol
ERα: Estrogen receptorα
ERβ: Estrogen receptorβ
FGF23: Fibroblast growth factor 23
GC-MS: Gas chromatography-mass spectrometry
GnRH: Gonadotropin-Releasing Hormone
GPER: G protein-coupled estrogen receptor
GPR30: G protein-coupled receptor 30
GO: Glycolate oxidase
GRHPR: Glyoxylate reductase
HK-2: Human proximal tubular epithelial cells
hRIFs: Human renal interstitial fibroblasts
KL: Klotho gene
LDH: Lactate dehydrogenase
MGP: Matrix Gla protein
mtROS: Mitochondrial reactive oxygen species
NHANES: National Health and Nutrition Examination Survey
NHS I: Nurses’ Health Study I
NCX1: Sodium-calcium exchanger 1
NaPi-IIa/IIb/IIc: Type IIa/IIb/IIc sodium-phosphate cotransporter
OPN: Osteopontin
PTH: Parathyroid hormone
PH: Primary hyperoxaluria
PMCA: Plasma membrane calcium-ATPase
PT: Proximal tubule
RTEC: Renal tubular epithelial cell
SERMs: Selective estrogen receptor modulators
SIRT3: Sirtuin 3
TAL: Thick ascending limb of the loop of Henle
TSECs: Tissue-selective estrogen complexes
TRPV5: Transient receptor potential vanilloid channel 5
VK: Von Kossa
VSMC: Vascular smooth muscle cell
WHI: Women’s Health Initiative

## References

[1] Wang K, et al. Risk factors for kidney stone disease recurrence: a comprehensive meta-analysis. BMC Urol. 2022;22(1):62. 35439979 10.1186/s12894-022-01017-4 PMC9017041

[2] Ramaswamy K, et al. The elementome of calcium-based urinary stones and its role in urolithiasis. Nat Rev Urol. 2015;12(10):543–57. 26334088 10.1038/nrurol.2015.208 PMC4875766

[3] Bishop K, Momah T, Ricks J. Nephrolithiasis Prim Care. 2020;47(4):661–71. 33121635 10.1016/j.pop.2020.08.005

[4] Sayer JA. Progress in understanding the genetics of calcium-containing nephrolithiasis. J Am Soc Nephrol. 2017;28(3):748–59. 27932479 10.1681/ASN.2016050576 PMC5328168

[5] Bargagli M, et al. Kidney stone disease: risk factors, pathophysiology and management. Nat Rev Nephrol. 2025;21(11):794–808. 40790363 10.1038/s41581-025-00990-x

[6] Chu L, et al. Associations between short-term temperature exposure and kidney-related conditions in new York state: the influence of temperature metrics across four dimensions. Environ Int. 2023;173:107783. 36841184 10.1016/j.envint.2023.107783

[7] Duan Q, et al. Association between composite dietary antioxidant index and kidney stone prevalence in adults: data from National health and nutrition examination survey (NHANES, 2007–2018). Front Nutr. 2024;11:1389714. 38840700 10.3389/fnut.2024.1389714 PMC11150772

[8] Khan SR, Canales BK, Dominguez-Gutierrez PR. Randall’s plaque and calcium oxalate stone formation: role for immunity and inflammation. Nat Rev Nephrol. 2021;17(6):417–33. 33514941 10.1038/s41581-020-00392-1

[9] Singh P, et al. The genetics of kidney stone disease and nephrocalcinosis. Nat Rev Nephrol. 2022;18(4):224–40. 34907378 10.1038/s41581-021-00513-4

[10] Ferraro PM, Taylor EN, Curhan GC. Factors associated with sex differences in the risk of nephrolithiasis. Nephrol Dial Transplant. 2023;38(1):177–83. 35138394 10.1093/ndt/gfac037 PMC9869853

[11] Abufaraj M, et al. Prevalence and trends in kidney stone among adults in the USA: analyses of National health and nutrition examination survey 2007–2018 data. Eur Urol Focus. 2021;7(6):1468–75. 32900675 10.1016/j.euf.2020.08.011

[12] Tundo G, Khaleel S, Pais VM Jr. Gender equivalence in the prevalence of nephrolithiasis among adults younger than 50 years in the united States. J Urol. 2018;200(6):1273–7. 30059688 10.1016/j.juro.2018.07.048

[13] Scales CD Jr., et al. Changing gender prevalence of stone disease. J Urol. 2007;177(3):979–82. 17296391 10.1016/j.juro.2006.10.069

[14] Xu JZ, et al. Sex disparities and the risk of urolithiasis: a large cross-sectional study. Ann Med. 2022;54(1):1627–35. 35675329 10.1080/07853890.2022.2085882 PMC9196832

[15] Sanchez BN, et al. Sex differences in energy metabolism: A Female-Oriented discussion. Sports Med. 2024;54(8):2033–57. 38888855 10.1007/s40279-024-02063-8

[16] Ferraro PM, et al. Dietary and lifestyle risk factors associated with incident nephrolithiasis in men and women. J Urol. 2017;198(4):858–63. 28365271 10.1016/j.juro.2017.03.124 PMC5599330

[17] Khalil R, et al. Sex steroids and the kidney: role in renal calcium and phosphate handling. Mol Cell Endocrinol. 2018;465:61–72. 29155307 10.1016/j.mce.2017.11.011

[18] Yagisawa T, et al. The influence of sex hormones on renal osteopontin expression and urinary constituents in experimental urolithiasis. J Urol. 2001;166(3):1078–82. 11490302

[19] Fleisch H. Inhibitors and promoters of stone formation. Kidney Int. 1978;13(5):361–71. 351264 10.1038/ki.1978.54

[20] Prochaska M, et al. Relative supersaturation of 24-Hour urine and likelihood of nephrolithiasis. J Urol. 2018;199(5):1262–6. 29132983 10.1016/j.juro.2017.10.046 PMC5911189

[21] Pak CY. Physicochemical basis for formation of renal stones of calcium phosphate origin: calculation of the degree of saturation of urine with respect to brushite. J Clin Invest. 1969;48(10):1914–22. 5822595 10.1172/JCI106158 PMC322428

[22] Parks JH, Coward M, Coe FL. Correspondence between stone composition and urine supersaturation in nephrolithiasis. Kidney Int. 1997;51(3):894–900. 9067927 10.1038/ki.1997.126

[23] Menon M, Mahle CJ. Oxalate metabolism and renal calculi. J Urol. 1982;127(1):148–51. 7035692 10.1016/s0022-5347(17)53649-6

[24] Mitchell T, et al. Dietary oxalate and kidney stone formation. Am J Physiol Ren Physiol. 2019;316(3):F409–13. 10.1152/ajprenal.00373.2018 PMC6459305 30566003

[25] Noonin C, Thongboonkerd V. Beneficial roles of Gastrointestinal and urinary microbiomes in kidney stone prevention via their oxalate-degrading ability and beyond. Microbiol Res. 2024;282:127663. 38422861 10.1016/j.micres.2024.127663

[26] Hesse A, Heimbach D. Causes of phosphate stone formation and the importance of metaphylaxis by urinary acidification: a review. World J Urol. 1999;17(5):308–15. 10552150 10.1007/s003450050152

[27] Guimerà J, et al. Prevalence of distal renal tubular acidosis in patients with calcium phosphate stones. World J Urol. 2020;38(3):789–94. 31079188 10.1007/s00345-019-02804-9

[28] Arcidiacono T, et al. Idiopathic calcium nephrolithiasis: a review of pathogenic mechanisms in the light of genetic studies. Am J Nephrol. 2014;40(6):499–506. 25504362 10.1159/000369833

[29] Xu Z, et al. Metabolic changes in kidney stone disease. Front Immunol. 2023;14:1142207. 37228601 10.3389/fimmu.2023.1142207 PMC10203412

[30] Khan SR, et al. Nephrolithiasis Nat Rev Dis Primers. 2016;2:16008. 27188687 10.1038/nrdp.2016.8 PMC5685519

[31] Curry JN, Yu ASL. Paracellular calcium transport in the proximal tubule and the formation of nephrolithiasis. Am J Physiol Renal Physiol. 2019;316(5):F966-9. 30838875 10.1152/ajprenal.00519.2018 PMC6580246

[32] Bargagli M, Ferraro PM, Vittori M, Lombardi G, Gambaro G, Somani B. Calcium and Vitamin D supplementation and their association with kidney stone disease: A narrative review. Nutrients. 2021; 13(12):4363. 10.3390/nu13124363 PMC8707627 34959915

[33] Ko B, et al. Sex differences in proximal and distal nephron function contribute to the mechanism of idiopathic hypercalcuria in calcium stone formers. Am J Physiol Regul Integr Comp Physiol. 2015;309(1):R85–92. 25947170 10.1152/ajpregu.00071.2015 PMC4491535

[34] Ganesan C, et al. Analysis of primary hyperparathyroidism screening among US veterans with nephrolithiasis. JAMA Surg. 2020;155(9):861–8. 32725208 10.1001/jamasurg.2020.2423 PMC7391180

[35] Alexander RT, Fuster DG, Dimke H. Mechanisms Underlying Calcium Nephrolithiasis Annu Rev Physiol. 2022;84:559–83. 34699268 10.1146/annurev-physiol-052521-121822

[36] Letavernier E, Daudon M. Vitamin D, Hypercalciuria and Kidney Stones. Nutrients. 2018;10(3):366. 10.3390/nu10030366 PMC5872784 29562593

[37] Chen T, et al. Oxalate as a potent promoter of kidney stone formation. Front Med (Lausanne). 2023;10:1159616. 37342493 10.3389/fmed.2023.1159616 PMC10278359

[38] Aronson PS. Role of SLC26A6-mediated Cl⁻-oxalate exchange in renal physiology and pathophysiology. J Nephrol. 2010;23:S158–64. 21170874

[39] Brzica H, et al. Oxalate: from the environment to nephrolithiasis. Arh Hig Rada Toksikol. 2013;64(4):609–30. 24384768 10.2478/10004-1254-64-2013-2428

[40] Gee HY, et al. Mutations in SLC26A1 cause nephrolithiasis. Am J Hum Genet. 2016;98(6):1228–34. 27210743 10.1016/j.ajhg.2016.03.026 PMC4908148

[41] Whittamore JM, Stephens CE, Hatch M. Absence of the sulfate transporter SAT-1 has no impact on oxalate handling by mouse intestine and does not cause hyperoxaluria or hyperoxalemia. Am J Physiol Gastrointest Liver Physiol. 2019;316(1):G82–94. 30383413 10.1152/ajpgi.00299.2018 PMC6383384

[42] Milliner DS, et al. End points for clinical trials in primary hyperoxaluria. Clin J Am Soc Nephrol. 2020;15(7):1056–65. 32165440 10.2215/CJN.13821119 PMC7341772

[43] Cochat P, Rumsby G. Primary hyperoxaluria. N Engl J Med. 2013;369(7):649–58. 23944302 10.1056/NEJMra1301564

[44] Fargue S, Acquaviva C, Bourdain. Primary hyperoxaluria type 1: pathophysiology and genetics. Clin Kidney J. 2022;15(Suppl 1):i4–8. 35592619 10.1093/ckj/sfab217 PMC9113437

[45] Wood K.D., Holmes R.P., Knight J. RNA interference in the treatment of renal stone disease: current status and future potentials. Int J Surg. 2016;36Pt D:713–6. 10.1016/j.ijsu.2016.11.027 PMC5218583 27847291

[46] Hopp K, et al. Phenotype-Genotype correlations and estimated carrier frequencies of primary hyperoxaluria. J Am Soc Nephrol. 2015;26(10):2559–70. 25644115 10.1681/ASN.2014070698 PMC4587693

[47] Danpure CJ, Rumsby G. Molecular aetiology of primary hyperoxaluria and its implications for clinical management. Expert Rev Mol Med. 2004;6(1):1–16. 14987413 10.1017/S1462399404007203

[48] Knight J, et al. Hydroxyproline metabolism in mouse models of primary hyperoxaluria. Am J Physiol Ren Physiol. 2012;302(6):F688–93. 10.1152/ajprenal.00473.2011 PMC3311317 22189945

[49] Singh P, et al. Primary hyperoxaluria type 3 can also result in kidney failure: A case report. Am J Kidney Dis. 2022;79(1):125–8. 34245816 10.1053/j.ajkd.2021.05.016 PMC8692335

[50] Fuster DG, et al. Association of urinary sex steroid hormones with urinary calcium, oxalate and citrate excretion in kidney stone formers. Nephrol Dial Transpl. 2022;37(2):335–48. 10.1093/ndt/gfaa360 33295624

[51] Karaica D, et al. Sex-independent expression of chloride/formate exchanger Cfex (Slc26a6) in rat pancreas, small intestine, and liver, and male-dominant expression in kidneys. Arh Hig Rada Toksikol. 2018;69(4):286–303. 30864378 10.2478/aiht-2018-69-3157

[52] Lee D, et al. Estrogen treatment reduced oxalate transporting activity and enhanced migration through the involvement of SLC26A6 in lung cancer cells. Toxicol Vitro. 2022;82:105373. 10.1016/j.tiv.2022.105373 35500753

[53] Jin H, et al. Oestrogen upregulates the expression levels and functional activities of duodenal mucosal CFTR and SLC26A6. Exp Physiol. 2016;101(11):1371–82. 27615377 10.1113/EP085803

[54] Tarhuni M, et al. Estrogen’s tissue-specific regulation of the SLC26A6 anion transporter reveal a phenotype of kidney stone disease in estrogen-deficient females: a systematic review. Cureus. 2023;15(9):e45839. 37881392 10.7759/cureus.45839 PMC10597593

[55] Brzica H, et al. The liver and kidney expression of sulfate anion transporter sat-1 in rats exhibits male-dominant gender differences. Pflugers Arch. 2009;457(6):1381–92. 19002488 10.1007/s00424-008-0611-5

[56] Yoshioka I, et al. Effect of sex hormones on crystal formation in a stone-forming rat model. Urology. 2010;75(4):907–13. 20163845 10.1016/j.urology.2009.09.094

[57] Breljak D, et al. In female rats, ethylene glycol treatment elevates protein expression of hepatic and renal oxalate transporter sat-1 (Slc26a1) without inducing hyperoxaluria. Croat Med J. 2015;56(5):447–59. 26526882 10.3325/cmj.2015.56.447 PMC4655930

[58] Liu Y, et al. Protective effect of estradiol on hepatocytic oxidative damage. World J Gastroenterol. 2002;8(2):363–6. 11925626 10.3748/wjg.v8.i2.363 PMC4658385

[59] Liang L, et al. Androgen receptor enhances kidney stone-CaOx crystal formation via modulation of oxalate biosynthesis & oxidative stress. Mol Endocrinol. 2014;28(8):1291–303. 24956378 10.1210/me.2014-1047 PMC4116591

[60] Zhu W, et al. The protective roles of Estrogen receptor β in renal calcium oxalate crystal formation via reducing the liver oxalate biosynthesis and renal oxidative Stress-Mediated cell injury. Oxid Med Cell Longev. 2019;2019:p5305014. 10.1155/2019/5305014 PMC6501165 31178964

[61] Randall A. The origin and growth of renal calculi. Ann Surg. 1937;105(6):1009–27. 17856988 10.1097/00000658-193706000-00014 PMC1390483

[62] Sepe V, et al. Henle loop basement membrane as initial site for Randall plaque formation. Am J Kidney Dis. 2006;48(5):706–11. 17059989 10.1053/j.ajkd.2006.07.021

[63] Murray SL, Wolf M. Calcium and phosphate disorders: core curriculum 2024. Am J Kidney Dis. 2024;83(2):241–56. 38099870 10.1053/j.ajkd.2023.04.017

[64] Michigami T, et al. Phosphate as a signaling molecule and its sensing mechanism. Physiol Rev. 2018;98(4):2317–48. 30109818 10.1152/physrev.00022.2017

[65] Hernando N, Wagner CA. Mechanisms and regulation of intestinal phosphate absorption. Compr Physiol. 2018;8(3):1065–90. 29978897 10.1002/cphy.c170024

[66] Biber J, Hernando N, Forster I. Phosphate transporters and their function. Annu Rev Physiol. 2013;75:535–50. 23398154 10.1146/annurev-physiol-030212-183748

[67] Segawa H, et al. Npt2a and Npt2c in mice play distinct and synergistic roles in inorganic phosphate metabolism and skeletal development. Am J Physiol Ren Physiol. 2009;297(3):F671–8. 10.1152/ajprenal.00156.2009 19570882

[68] Khan SR, Glenton PA. Calcium oxalate crystal deposition in kidneys of hypercalciuric mice with disrupted type IIa sodium-phosphate cotransporter. Am J Physiol Renal Physiol. 2008;294(5):F1109-15. 18337544 10.1152/ajprenal.00620.2007 PMC3625965

[69] Tatsumi S, et al. Regulation of renal phosphate handling: inter-organ communication in health and disease. J Bone Min Metab. 2016;34(1):1–10. 10.1007/s00774-015-0705-z 26296817

[70] Du C, et al. Renal Klotho and inorganic phosphate are extrinsic factors that antagonistically regulate hematopoietic stem cell maintenance. Cell Rep. 2022;38(7):110392. 35172146 10.1016/j.celrep.2022.110392

[71] Xu Y, Sun Z. Molecular basis of klotho: from gene to function in aging. Endocr Rev. 2015;36(2):174–93. 25695404 10.1210/er.2013-1079 PMC4399270

[72] Ahmatjan B, et al. Klotho inhibits the formation of calcium oxalate stones by regulating the Keap1-Nrf2-ARE signaling pathway. Int Urol Nephrol. 2023;55(2):263–76. 36336747 10.1007/s11255-022-03398-9

[73] Xiao Y, Xiao Z. Association between serum Klotho and nephrolithiasis in US middle-aged and older individuals with diabetes mellitus: results from 2007 to 2016 National health and nutrition survey. Am J Nephrol. 2023;54(5–6):224–33. 37231844 10.1159/000531045 PMC10614277

[74] Zhu Z, et al. α-Klotho released from HK-2 cells inhibits osteogenic differentiation of renal interstitial fibroblasts by inactivating the Wnt-β-catenin pathway. Cell Mol Life Sci. 2021;78(23):p7831–7849. 10.1007/s00018-021-03972-x PMC11071709 34724098

[75] Burnett-Bowie SM, Mendoza N, Leder BZ. Effects of gonadal steroid withdrawal on serum phosphate and FGF-23 levels in men. Bone. 2007;40(4):913–8. 17157573 10.1016/j.bone.2006.10.016 PMC2083121

[76] Wang C, et al. Effects of transdermal testosterone gel on bone turnover markers and bone mineral density in hypogonadal men. Clin Endocrinol (Oxf). 2001;54(6):739–50. 11422108 10.1046/j.1365-2265.2001.01271.x

[77] Pedersen L, et al. Reduction of calprotectin and phosphate during testosterone therapy in aging men: a randomized controlled trial. J Endocrinol Invest. 2017;40(5):529–38. 28000180 10.1007/s40618-016-0597-3

[78] Bosman A, et al. Sexual dimorphisms in serum calcium and phosphate concentrations in the Rotterdam study. Sci Rep. 2023;13(1):8310. 37221192 10.1038/s41598-023-34800-w PMC10205794

[79] Dick IM, et al. Effects of endogenous Estrogen on renal calcium and phosphate handling in elderly women. Am J Physiol Endocrinol Metab. 2005;288(2):E430–5. 15466921 10.1152/ajpendo.00140.2004

[80] Uemura H, et al. Close correlation between Estrogen treatment and renal phosphate reabsorption capacity. J Clin Endocrinol Metab. 2000;85(3):1215–9. 10720065 10.1210/jcem.85.3.6456

[81] Burris D, et al. Estrogen directly and specifically downregulates NaPi-IIa through the activation of both Estrogen receptor isoforms (ERα and ERβ) in rat kidney proximal tubule. Am J Physiol Ren Physiol. 2015;308(6):F522–34. 10.1152/ajprenal.00386.2014 PMC4360034 25608964

[82] Faroqui S, et al. Estrogen downregulates the proximal tubule type IIa sodium phosphate cotransporter causing phosphate wasting and hypophosphatemia. Kidney Int. 2008;73(10):1141–50. 18305465 10.1038/ki.2008.33 PMC2738940

[83] Webster R, et al. Klotho/fibroblast growth factor 23- and PTH-independent Estrogen receptor-α-mediated direct downregulation of NaPi-IIa by Estrogen in the mouse kidney. Am J Physiol Ren Physiol. 2016;311(2):F249–59. 10.1152/ajprenal.00542.2015 PMC5008677 27194721

[84] Zhang D, et al. Effects of sex and postmenopausal Estrogen use on serum phosphorus levels: a cross-sectional study of the National health and nutrition examination survey (NHANES) 2003–2006. Am J Kidney Dis. 2014;63(2):198–205. 24051078 10.1053/j.ajkd.2013.07.012

[85] Adomako EA, et al. Urine pH and citrate as predictors of calcium phosphate stone formation. Kidney360. 2023;4(8):1123–9. 37307531 10.34067/KID.0000000000000184 PMC10476682

[86] Awuah Boadi E, et al. Sex-specific Stone-forming phenotype in mice during Hypercalciuria/Urine alkalinization. Lab Invest. 2024;104(5):102047. 38452902 10.1016/j.labinv.2024.102047 PMC11103239

[87] Parks JH, et al. Clinical implications of abundant calcium phosphate in routinely analyzed nephrolithiasis. Kidney Int. 2004;66(2):777–85. 15253733 10.1111/j.1523-1755.2004.00803.x

[88] Zhang Z, et al. Association between testosterone and serum soluble α-klotho in U.S. Males: a cross-sectional study. BMC Geriatr. 2022;22(1):570. 35820842 10.1186/s12877-022-03265-3 PMC9275159

[89] Tippen SP, et al. Age and sex effects on FGF23-mediated response to mild phosphate challenge. Bone. 2021;146:115885. 33618073 10.1016/j.bone.2021.115885 PMC8009839

[90] Adams-Sherrod GA, Brooks HL, Kumar P. Sex-specific modulation of renal epigenetic and injury markers in aging kidney. Am J Physiol-Renal Physiol. 2024;327(3):F543-51. 38961843 10.1152/ajprenal.00140.2024 PMC11460336

[91] Yang W, et al. Association of kidney disease outcomes with risk factors for CKD: findings from the chronic renal insufficiency cohort (CRIC) study. Am J Kidney Dis. 2014;63(2):236–43. 24182662 10.1053/j.ajkd.2013.08.028 PMC3946885

[92] Ohnishi M, Razzaque MS. Dietary and genetic evidence for phosphate toxicity accelerating mammalian aging. Faseb J. 2010;24(9):3562–71. 20418498 10.1096/fj.09-152488 PMC2923352

[93] Kuro-o M. Klotho, phosphate and FGF-23 in ageing and disturbed mineral metabolism. Nat Rev Nephrol. 2013;9(11):650–60. 23774819 10.1038/nrneph.2013.111

[94] Kuro OM. A phosphate-centric paradigm for pathophysiology and therapy of chronic kidney disease. Kidney Int Suppl (2011). 2013;3(5):420–6. 25019024 10.1038/kisup.2013.88 PMC4089674

[95] Chen K, Sun Z. Estrogen inhibits renal Na-Pi Co-transporters and improves Klotho deficiency-induced acute heart failure. Redox Biol. 2021;47:102173. 34678656 10.1016/j.redox.2021.102173 PMC8577443

[96] Alexander RT, et al. Nephrolithiasis and cardiovascular events: a cohort study. Clin J Am Soc Nephrol. 2014;9(3):506–12. 24311706 10.2215/CJN.04960513 PMC3944758

[97] Muschialli L, et al. Epidemiological and biological associations between cardiovascular disease and kidney stone formation: A systematic review and meta-analysis. Nutr Metab Cardiovasc Dis. 2024;34(3):559–68. 38431384 10.1016/j.numecd.2023.09.011

[98] Yu ASL, Curry JN. Paracellular transport and renal tubule calcium handling: emerging roles in kidney stone disease. J Am Soc Nephrol. 2024;35(12):1758–67. 39207856 10.1681/ASN.0000000506 PMC11617488

[99] Alexander RT, Dimke H. Molecular mechanisms underlying paracellular calcium and magnesium reabsorption in the proximal tubule and thick ascending limb. Ann N Y Acad Sci. 2022;1518(1):69–83. 36200584 10.1111/nyas.14909

[100] Subramanya AR, Ellison DH. Distal convoluted tubule. Clin J Am Soc Nephrol. 2014;9(12):2147–63. 24855283 10.2215/CJN.05920613 PMC4255408

[101] Magyar CE, et al. Plasma membrane Ca2+-ATPase and NCX1 Na+/Ca2 + exchanger expression in distal convoluted tubule cells. Am J Physiol Ren Physiol. 2002;283(1):F29–40. 10.1152/ajprenal.00252.2000 12060584

[102] Hoenderop JG, Nilius B, Bindels RJ. Calcium absorption across epithelia. Physiol Rev. 2005;85(1):373–422. 15618484 10.1152/physrev.00003.2004

[103] Woudenberg-Vrenken TE, Bindels RJ, Hoenderop JG. The role of transient receptor potential channels in kidney disease. Nat Rev Nephrol. 2009;5(8):441–9. 19546862 10.1038/nrneph.2009.100

[104] Guleray Lafci N, et al. Decreased calcium permeability caused by biallelic TRPV5 mutation leads to autosomal recessive renal calcium-wasting hypercalciuria. Eur J Hum Genet. 2024;32(11):1506–14. 38528055 10.1038/s41431-024-01589-9 PMC11577068

[105] van der Wijst J, et al. TRPV5 in renal tubular calcium handling and its potential relevance for nephrolithiasis. Kidney Int. 2019;96(6):1283–91. 31471161 10.1016/j.kint.2019.05.029

[106] Bove-Fenderson E, Mannstadt M. Hypocalcemic disorders. Best Pract Res Clin Endocrinol Metab. 2018;32(5):639–56. 30449546 10.1016/j.beem.2018.05.006

[107] Pasch A, et al. PTH and 1.25 vitamin D response to a low-calcium diet is associated with bone mineral density in renal stone formers. Nephrol Dial Transpl. 2008;23(8):2563–70. 10.1093/ndt/gfn091 18398019

[108] Kim WT, et al. Role of 1,25-dihydroxy vitamin D3 and parathyroid hormone in urinary calcium excretion in calcium stone formers. Yonsei Med J. 2014;55(5):1326–32. 25048492 10.3349/ymj.2014.55.5.1326 PMC4108819

[109] Shakhssalim N, et al. An assessment of parathyroid hormone, calcitonin, 1,25 (OH)2 vitamin D3, estradiol and testosterone in men with active calcium stone disease and evaluation of its biochemical risk factors. Urol Res. 2011;39(1):1–7. 20490785 10.1007/s00240-010-0276-3

[110] Malihi Z, et al. Hypercalcemia, hypercalciuria, and nephrolithiasis in long-term studies of vitamin D supplementation: a systematic review and meta-analysis. Am J Clin Nutr. 2016;104(4):1039–51. 27604776 10.3945/ajcn.116.134981

[111] Letavernier E, et al. Calcium and vitamin D have a synergistic role in a rat model of kidney stone disease. Kidney Int. 2016;90(4):809–17. 27475231 10.1016/j.kint.2016.05.027

[112] Ticinesi A, et al. Idiopathic calcium nephrolithiasis and hypovitaminosis D: A Case-control study. Urology. 2016;87:40–5. 26494294 10.1016/j.urology.2015.10.009

[113] Girón-Prieto MS, et al. Analysis of vitamin D deficiency in calcium stone-forming patients. Int Urol Nephrol. 2016;48(8):1243–6. 27093967 10.1007/s11255-016-1290-3

[114] Zhang F, Li W. The complex relationship between vitamin D and nephrolithiasis: balance, risks, and prevention strategies. Front Nutr. 2024;11:1435403. 39346653 10.3389/fnut.2024.1435403 PMC11427370

[115] Portales-Castillo I, Simic P. PTH, FGF-23, Klotho and vitamin D as regulators of calcium and phosphorus: genetics, epigenetics and beyond. Front Endocrinol (Lausanne). 2022;13:992666. 36246903 10.3389/fendo.2022.992666 PMC9558279

[116] Mitra P, et al. Polymorphisms of PTH (Parathyroid Hormone) gene and risk of kidney stone disease: A Case-Control study from West Bengal, India. Urology. 2018;121:79–85. 29969593 10.1016/j.urology.2018.06.033

[117] Minisola S, et al. Classical complications of primary hyperparathyroidism. Best Pract Res Clin Endocrinol Metab. 2018;32(6):791–803. 30665547 10.1016/j.beem.2018.09.001

[118] Suh JM, Cronan JJ, Monchik JM. Primary hyperparathyroidism: is there an increased prevalence of renal stone disease? AJR Am J Roentgenol. 2008;191(3):908–11. 18716127 10.2214/AJR.07.3160

[119] Zabolotniuk T, et al. Screening for asymptomatic nephrolithiasis in primary hyperparathyroidism patients is warranted. Am J Surg. 2024;231:91–5. 38480062 10.1016/j.amjsurg.2024.03.007

[120] Oz OK, et al. Aromatase deficiency causes altered expression of molecules critical for calcium reabsorption in the kidneys of female mice *. J Bone Min Res. 2007;22(12):1893–902. 10.1359/jbmr.070808 17708714

[121] Van Abel M, Hoenderop JG, Dardenne O, St Arnaud R, Van Os CH, Van Leeuwen HJ, Bindels RJ, 1,25-dihydroxyvitaminD(3)-independent stimulatory effect of estrogen on the expression of ECaC1 in the kidney. J Am Soc Nephrol. 2002;13:2102–2109. 10.1097/01.asn.0000022423.34922.2a 12138142

[122] Dong XL, Zhang Y, Wong MS. Estrogen deficiency-induced Ca balance impairment is associated with decrease in expression of epithelial Ca transport proteins in aged female rats. Life Sci. 2014;96(1–2):26–32. 24378673 10.1016/j.lfs.2013.12.025

[123] Jackson RD, et al. Calcium plus vitamin D supplementation and the risk of fractures. N Engl J Med. 2006;354(7):669–83. 16481635 10.1056/NEJMoa055218

[124] Curhan GC, et al. Comparison of dietary calcium with supplemental calcium and other nutrients as factors affecting the risk for nephrolithiasis in women. Ann Intern Med. 1997;126(7):497–504. 9092314 10.7326/0003-4819-126-7-199704010-00001

[125] Curhan GC, et al. A prospective study of dietary calcium and other nutrients and the risk of symptomatic nephrolithiasis. N Engl J Med. 1993;328(12):833–8. 8441427 10.1056/NEJM199303253281203

[126] Borghi L, et al. Comparison of two diets for the prevention of recurrent stones in idiopathic hypercalciuria. N Engl J Med. 2002;346(2):77–84. 11784873 10.1056/NEJMoa010369

[127] Yang Y, et al. Efficacy of combination therapy of vitamin D and bisphosphonates in the treatment of postmenopausal osteoporosis: a systematic review and meta-analysis. Front Pharmacol. 2024;15:1422062. 39640483 10.3389/fphar.2024.1422062 PMC11617160

[128] Prochaska M, Taylor EN, Curhan G. Menopause and risk of nephrolithiasis. J Urol. 2018;200(4):823–8. 29730204 10.1016/j.juro.2018.04.080 PMC6556766

[129] Ferraro PM, et al. Vitamin D intake and the risk of incident nephrolithiasis. J Urol. 2017;197(2):405–10. 27545576 10.1016/j.juro.2016.08.084 PMC5241241

[130] Safari H, et al. Consequences of vitamin D deficiency or overdosage on follicular development and steroidogenesis in Normo and hypo calcemic mouse models. Sci Rep. 2025;15(1):14278. 40274992 10.1038/s41598-025-99437-3 PMC12022079

[131] Tang TY, et al. The association between menopause, postmenopausal hormone therapy, and kidney stone disease in Taiwanese women. Ann Epidemiol. 2023;78:13–8. 36494042 10.1016/j.annepidem.2022.12.002

[132] Barrionuevo P, et al. Efficacy of Pharmacological therapies for the prevention of fractures in postmenopausal women: A network Meta-Analysis. J Clin Endocrinol Metab. 2019;104(5):1623–30. 30907957 10.1210/jc.2019-00192

[133] Bolognese MA. SERMs and serms with estrogen for postmenopausal osteoporosis. Rev Endocr Metab Disord. 2010;11(4):253–9. 20446043 10.1007/s11154-010-9137-1

[134] Pinkerton JV, Stovall DW. Bazedoxifene when paired with conjugated estrogens is a new paradigm for treatment of postmenopausal women. Expert Opin Investig Drugs. 2010;19(12):1613–21. 21073353 10.1517/13543784.2010.532487

[135] Li JY, et al. Testosterone and androgen receptor in human nephrolithiasis. J Urol. 2010;184(6):2360–3. 20952021 10.1016/j.juro.2010.08.009

[136] Sharma JK, et al. Serum total testosterone is associated with phosphate and calcium excretion in response to oral phosphate loading in healthy middle-aged males. Andrology. 2024;12(8):1668–74. 38376008 10.1111/andr.13607

[137] Hsu YJ, et al. Testosterone increases urinary calcium excretion and inhibits expression of renal calcium transport proteins. Kidney Int. 2010;77(7):601–8. 20090667 10.1038/ki.2009.522

[138] Dick IM, et al. Estrogen and androgen regulation of plasma membrane calcium pump activity in immortalized distal tubule kidney cells. Mol Cell Endocrinol. 2003;212(1–2):11–8. 14654246 10.1016/j.mce.2003.09.028

[139] Ågmo A. Androgen receptors and sociosexual behaviors in mammals: the limits of generalization. Neurosci Biobehav Rev. 2024;157:105530. 38176634 10.1016/j.neubiorev.2023.105530

[140] Naamneh Elzenaty R, Toit Tdu, Flück CE. Basics of androgen synthesis and action. Best Pract Res Clin Endocrinol Metab. 2022;36(4):101665. 35595638 10.1016/j.beem.2022.101665

[141] Sueksakit K, Thongboonkerd V. Protective effects of finasteride against testosterone-induced calcium oxalate crystallization and crystal-cell adhesion. J Biol Inorg Chem. 2019;24(7):973–83. 31342142 10.1007/s00775-019-01692-z

[142] Lin PH, et al. Induction of renal senescence marker protein-30 (SMP30) expression by testosterone and its contribution to urinary calcium absorption in male rats. Sci Rep. 2016;6:32085. 27553527 10.1038/srep32085 PMC4995462

[143] Gaumet-Meunier N, et al. Gonadal steroids and bone metabolism in young castrated male rats. Calcif Tissue Int. 2000;66(6):470–5. 10821886 10.1007/s002230010094

[144] Khalil R, et al. Androgen action on renal calcium and phosphate handling: effects of bisphosphonate treatment and low calcium diet. Mol Cell Endocrinol. 2020;514:110891. 32533994 10.1016/j.mce.2020.110891

[145] Olmos-Ortiz A, et al. Evidence of sexual dimorphism in placental vitamin D metabolism: testosterone inhibits calcitriol-dependent Cathelicidin expression. J Steroid Biochem Mol Biol. 2016;163:173–82. 27210415 10.1016/j.jsbmb.2016.05.017

[146] Di X, et al. Association between the systemic immune-inflammation index and kidney stone: A cross-sectional study of NHANES 2007–2018. Front Immunol. 2023;14:1116224. 36895572 10.3389/fimmu.2023.1116224 PMC9989007

[147] Thongboonkerd V, Yasui T, Khan SR. Editorial: immunity and inflammatory response in kidney stone disease. Front Immunol. 2021;12:795559. 34790209 10.3389/fimmu.2021.795559 PMC8591093

[148] Mulay SR, Evan A, Anders HJ. Molecular mechanisms of crystal-related kidney inflammation and injury. Implications for cholesterol embolism, crystalline nephropathies and kidney stone disease. Nephrol Dial Transplant. 2014;29(3):507–14. 24163269 10.1093/ndt/gft248

[149] Sun Y, et al. New insight into oxidative stress and inflammatory responses to nephrolithiasis: potential therapeutic strategies with natural active ingredients. Biomed Pharmacother. 2024;179:117333. 39243436 10.1016/j.biopha.2024.117333

[150] Peerapen P, Thongboonkerd V. Caffeine prevents kidney stone formation by translocation of apical surface Annexin A1 crystal-binding protein into cytoplasm: in vitro evidence. Sci Rep. 2016;6:38536. 27924845 10.1038/srep38536 PMC5141452

[151] Fong-Ngern K, Thongboonkerd V. Alpha-enolase on apical surface of renal tubular epithelial cells serves as a calcium oxalate crystal receptor. Sci Rep. 2016;6:36103. 27796334 10.1038/srep36103 PMC5086859

[152] Khan SR. Reactive oxygen species as the molecular modulators of calcium oxalate kidney stone formation: evidence from clinical and experimental investigations. J Urol. 2013;189(3):803–11. 23022011 10.1016/j.juro.2012.05.078 PMC5683176

[153] Khan SR. Reactive oxygen species, inflammation and calcium oxalate nephrolithiasis. Transl Androl Urol. 2014;3(3):256–76. 25383321 10.3978/j.issn.2223-4683.2014.06.04 PMC4220551

[154] Taguchi K, et al. Macrophage function in calcium oxalate kidney stone formation: A systematic review of literature. Front Immunol. 2021;12:673690. 34108970 10.3389/fimmu.2021.673690 PMC8182056

[155] Liu J, et al. Polydatin protects against calcium oxalate crystal-induced renal injury through the cytoplasmic/mitochondrial reactive oxygen species-NLRP3 inflammasome pathway. Biomed Pharmacother. 2023;167:115621. 37793278 10.1016/j.biopha.2023.115621

[156] Chaiyarit S, Thongboonkerd V. Mitochondrial dysfunction and kidney stone disease. Front Physiol. 2020;11:566506. 33192563 10.3389/fphys.2020.566506 PMC7606861

[157] Su B, et al. Mitochondrial dysfunction and NLRP3 inflammasome: key players in kidney stone formation. BJU Int. 2024;134(5):696–713. 38967108 10.1111/bju.16454

[158] Ming S, et al. Oxalate-induced apoptosis through ERS-ROS-NF-κB signalling pathway in renal tubular epithelial cell. Mol Med. 2022;28(1):88. 35922749 10.1186/s10020-022-00494-5 PMC9347104

[159] Liang F, et al. Research progress on the mechanisms of Interleukin and chemokine families in driving calcium oxalate nephrolithiasis formation. Front Immunol. 2025;16:1651003. 41019076 10.3389/fimmu.2025.1651003 PMC12463896

[160] Ma HY, Chen S, Du Y. Estrogen and estrogen receptors in kidney diseases. Ren Fail. 2021;43(1):619–42. 33784950 10.1080/0886022X.2021.1901739 PMC8018493

[161] Chen P, Li B, Ou-Yang L. Role of Estrogen receptors in health and disease. Front Endocrinol (Lausanne). 2022;13:839005. 36060947 10.3389/fendo.2022.839005 PMC9433670

[162] Arao Y, Korach KS. The physiological role of estrogen receptor functional domains. Essays Biochem. 2021;65(6):867–75. 34028522 10.1042/EBC20200167 PMC8611119

[163] Matsuda T, et al. Cross-talk between transforming growth factor-beta and Estrogen receptor signaling through Smad3. J Biol Chem. 2001;276(46):42908–14. 11555647 10.1074/jbc.M105316200

[164] Cerillo G, et al. The oestrogen receptor regulates NFkappaB and AP-1 activity in a cell-specific manner. J Steroid Biochem Mol Biol. 1998;67(2):79–88. 9877207 10.1016/s0960-0760(98)00078-8

[165] Chin M, et al. Estrogen and raloxifene, a selective Estrogen receptor modulator, ameliorate renal damage in db/db mice. Am J Pathol. 2005;166(6):1629–36. 15920148 10.1016/s0002-9440(10)62473-x PMC1602422

[166] Kurt AH, et al. The protective role of G protein-coupled Estrogen receptor 1 (GPER-1) on methotrexate-induced nephrotoxicity in human renal epithelium cells. Ren Fail. 2016;38(5):686–92. 26981789 10.3109/0886022X.2016.1155398

[167] Hassan E, et al. The potential protective effects of estradiol and 2-methoxyestradiol in ischemia reperfusion-induced kidney injury in ovariectomized female rats. Life Sci. 2022;296:120441. 35240160 10.1016/j.lfs.2022.120441

[168] Özdemir Kumral ZN, et al. Estrogen receptor agonists alleviate cardiac and renal oxidative injury in rats with renovascular hypertension. Clin Exp Hypertens. 2016;38(6):500–9. 27399230 10.3109/10641963.2015.1116550

[169] Peerapen P, Thongboonkerd V. Protective cellular mechanism of estrogen against kidney stone formation: a proteomics approach and functional validation. Proteomics. 2019;19(19):e1900095. 31475403 10.1002/pmic.201900095

[170] Gottlieb B, et al. Nuclear receptors and disease: androgen receptor. Essays Biochem. 2004;40:121–36. 15242343 10.1042/bse0400121

[171] Allam S, et al. Androgen receptor Blockade by Flutamide down-regulates renal fibrosis, inflammation, and apoptosis pathways in male rats. Life Sci. 2023;323:121697. 37061126 10.1016/j.lfs.2023.121697

[172] Changtong C, et al. In vitro evidence of the promoting effect of testosterone in kidney stone disease: A proteomics approach and functional validation. J Proteom. 2016;144:11–22. 10.1016/j.jprot.2016.05.028 27260493

[173] Hu H, Zhou H, Xu D. A review of the effects and molecular mechanisms of Dimethylcurcumin (ASC-J9) on androgen receptor-related diseases. Chem Biol Drug Des. 2021;97(4):821–35. 33277796 10.1111/cbdd.13811

[174] Zhu W, et al. Loss of the androgen receptor suppresses intrarenal calcium oxalate crystals deposition via altering macrophage recruitment/M2 polarization with change of the miR-185-5p/CSF-1 signals. Cell Death Dis. 2019;10(4):275. 30894518 10.1038/s41419-019-1358-y PMC6427030

[175] van Eeghen SA, Pyle L, Narongkiatikhun P, Choi YJ, Obeid W, Parikh CR, Vosters TG, van Valkengoed IG, Krebber MM, Touw DJ, den Heijer M, Bjornstad P, van Raalte DH, Nokoff NJ. Unveiling mechanisms underlying kidney function changes during sex hormone therapy. J Clin Invest. 2025;135(9):e190850. 10.1172/JCI190850 PMC12043095 40193283

[176] Willems PH, et al. Redox homeostasis and mitochondrial dynamics. Cell Metab. 2015;22(2):207–18. 26166745 10.1016/j.cmet.2015.06.006

[177] Sultanova RF, et al. Sex differences in renal mitochondrial function: a hormone-gous opportunity for research. Am J Physiol Ren Physiol. 2020;319(6):F1117–24. 10.1152/ajprenal.00320.2020 PMC7792688 33135479

[178] Juszczak F, Arnould T, Declèves AE. The role of mitochondrial sirtuins (SIRT3, SIRT4 and SIRT5) in renal cell metabolism: Implication for kidney diseases. Int J Mol Sci. 2024;25(13):6936. 10.3390/ijms25136936 PMC11241570 39000044

[179] Yao H, et al. Sex-related differences in SIRT3-mediated mitochondrial dynamics in renal ischemia/reperfusion injury. Transl Res. 2024;270:1–12. 38556109 10.1016/j.trsl.2024.03.005

[180] Lin Q, et al. Inhibiting NLRP3 inflammasome attenuates apoptosis in contrast-induced acute kidney injury through the upregulation of HIF1A and BNIP3-mediated mitophagy. Autophagy. 2021;17(10):2975–90. 33345685 10.1080/15548627.2020.1848971 PMC8525960

[181] Fu ZJ, et al. HIF-1α-BNIP3-mediated mitophagy in tubular cells protects against renal ischemia/reperfusion injury. Redox Biol. 2020;36:101671. 32829253 10.1016/j.redox.2020.101671 PMC7452120

[182] Peng Y, et al. Testosterone induces renal tubular epithelial cell death through the HIF-1α/BNIP3 pathway. J Transl Med. 2019;17(1):62. 30819186 10.1186/s12967-019-1821-7 PMC6394048

[183] Verzola D, et al. Testosterone promotes apoptotic damage in human renal tubular cells. Kidney Int. 2004;65(4):1252–61. 15086464 10.1111/j.1523-1755.2004.00497.x

[184] Kusmartsev S, et al. Calcium oxalate stone fragment and crystal phagocytosis by human macrophages. J Urol. 2016;195(4 Pt 1):1143–51. 26626217 10.1016/j.juro.2015.11.048 PMC4882284

[185] Okada A, et al. Renal macrophage migration and crystal phagocytosis via inflammatory-related gene expression during kidney stone formation and elimination in mice: detection by association analysis of stone-related gene expression and microstructural observation. J Bone Min Res. 2010;25(12):2701–11. 10.1002/jbmr.158 20577968

[186] Zhu W, et al. Macrophage polarization regulation shed lights on immunotherapy for CaOx kidney stone disease. Biomed Pharmacother. 2024;179:117336. 39180792 10.1016/j.biopha.2024.117336

[187] Barcena ML, Christiansen-Mensch C, Aslam M, Haritonow N, Ladilov Y, Regitz-Zagrosek V. Upregulation of mitochondrial sirt3 and alleviation of the inflammatory phenotype in macrophages by estrogen. Cells. 2024;13(17):1420. 10.3390/cells13171420 PMC11393879 39272992

[188] Enright S, Werstuck GH. Investigating the effects of sex hormones on macrophage polarization. Int J Mol Sci. 2024;25(2):951. 10.3390/ijms25020951 PMC10816176 38256027

[189] Taguchi K, et al. Colony-stimulating factor-1 signaling suppresses renal crystal formation. J Am Soc Nephrol. 2014;25(8):1680–97. 24578130 10.1681/ASN.2013060675 PMC4116057

[190] Elshal AM, et al. Hormonal and molecular characterization of calcium oxalate stone formers predicting occurrence and recurrence. Urolithiasis. 2023;51(1):76. 37093310 10.1007/s00240-023-01440-8 PMC10125924

[191] Speer MY, et al. Inactivation of the osteopontin gene enhances vascular calcification of matrix Gla protein-deficient mice: evidence for osteopontin as an inducible inhibitor of vascular calcification in vivo. J Exp Med. 2002;196(8):1047–55. 12391016 10.1084/jem.20020911 PMC2194039

[192] Hsi RS, et al. Coronary artery calcium score and association with recurrent nephrolithiasis: the Multi-Ethnic study of atherosclerosis. J Urol. 2016;195(4 Pt 1):971–6. 26454103 10.1016/j.juro.2015.10.001 PMC4966606

[193] Vernon HJ, et al. Aprt/Opn double knockout mice: osteopontin is a modifier of kidney stone disease severity. Kidney Int. 2005;68(3):938–47. 16105024 10.1111/j.1523-1755.2005.00487.x

[194] Lee SJ, Lee IK, Jeon JH. Vascular calcification-new insights into its mechanism. Int J Mol Sci. 2020;21(8):2685. 10.3390/ijms21082685 PMC7216228 32294899

[195] Paloian NJ, Leaf EM, Giachelli CM. Osteopontin protects against high phosphate-induced nephrocalcinosis and vascular calcification. Kidney Int. 2016;89(5):1027–36. 27083280 10.1016/j.kint.2015.12.046 PMC4834144

[196] Miyajima J, et al. The interaction between female sex hormone receptors and osteopontin in a rat hyperoxaluric model. Kurume Med J. 2010;57(3):73–80. 21186342 10.2739/kurumemedj.57.73

[197] Sharafi MH, et al. The link between osteoporosis and cardiovascular diseases: a review of shared mechanisms, risk factors, and therapeutic approaches. Osteoporos Int. 2025;36(7):1129–42. 40455217 10.1007/s00198-025-07553-7

[198] Luo G, et al. Spontaneous calcification of arteries and cartilage in mice lacking matrix GLA protein. Nature. 1997;386(6620):78–81. 9052783 10.1038/386078a0

[199] Khavandgar Z, et al. Elastin haploinsufficiency impedes the progression of arterial calcification in MGP-deficient mice. J Bone Min Res. 2014;29(2):327–37. 10.1002/jbmr.2039 23857752

[200] Khan A, Wang W, Khan SR. Calcium oxalate nephrolithiasis and expression of matrix GLA protein in the kidneys. World J Urol. 2014;32(1):123–30. 23475213 10.1007/s00345-013-1050-2 PMC3731399

[201] Lu X, et al. A polymorphism of matrix Gla protein gene is associated with kidney stone in the Chinese Han population. Gene. 2012;511(2):127–30. 23046575 10.1016/j.gene.2012.09.112

[202] Osako MK, et al. Estrogen inhibits vascular calcification via vascular RANKL system: common mechanism of osteoporosis and vascular calcification. Circ Res. 2010;107(4):466–75. 20595654 10.1161/CIRCRESAHA.110.216846

[203] Mattix Kramer HJ, et al. Menopause and postmenopausal hormone use and risk of incident nephrolithiasis. J Am Soc Nephrol. 2003;14(5):1272–7. 12707395 10.1097/01.asn.0000060682.25472.c3

[204] Heller HJ, et al. Etiological role of Estrogen status in renal stone formation. J Urol. 2002;168(5):1923–7. 12394677 10.1016/S0022-5347(05)64264-4

[205] Lobo RA. Benefits and risks of estrogen replacement therapy. Am J Obstet Gynecol. 1995;173(3 Pt 2):982–9. 7573295 10.1016/0002-9378(95)90247-3

[206] Liu JH. Selective estrogen receptor modulators (SERMS): keys to understanding their function. Menopause. 2020;27(10):1171–6. 32576800 10.1097/GME.0000000000001585

[207] Stanczyk FZ, et al. Comparison of Estrogens and selective Estrogen receptor modulators (SERMs) used for menopausal hormone therapy. Menopause. 2025;32(8):730–57. 40742784 10.1097/GME.0000000000002547

[208] Lee WL, et al. The benefits of Estrogen or selective Estrogen receptor modulator on kidney and its related disease-chronic kidney disease-mineral and bone disorder: osteoporosis. J Chin Med Assoc. 2013;76(7):365–71. 23664736 10.1016/j.jcma.2013.03.010

[209] Hernandez P, et al. The selective Estrogen receptor Modulator, Raloxifene, is protective against renal ischemia-reperfusion injury. Transplantation. 2022;106(11):2166–71. 35655356 10.1097/TP.0000000000004194 PMC12063156

[210] Di Lorenzo G, Autorino R. *Bicalutamide-induced gynaecomastia: do we have the answer?* Eur Urol. 2007;52(1):5–8. 17258386 10.1016/j.eururo.2007.01.063

[211] Neyman A, Fuqua JS, Eugster EA. Bicalutamide as an androgen blocker with secondary effect of promoting feminization in male-to-female transgender adolescents. J Adolesc Health. 2019;64(4):544–6. 30612811 10.1016/j.jadohealth.2018.10.296 PMC6431559

[212] Smith MR, et al. Changes in body composition during androgen deprivation therapy for prostate cancer. J Clin Endocrinol Metab. 2002;87(2):599–603. 11836291 10.1210/jcem.87.2.8299

[213] He D, et al. ASC-J9 suppresses renal cell carcinoma progression by targeting an androgen receptor-dependent HIF2α/VEGF signaling pathway. Cancer Res. 2014;74(16):4420–30. 24924778 10.1158/0008-5472.CAN-13-2681

[214] Goleij P, et al. Unlocking daidzein’s healing power: present applications and future possibilities in phytomedicine. Phytomedicine. 2024;134:155949. 39217652 10.1016/j.phymed.2024.155949

[215] Patra S, et al. A review on phytoestrogens: current status and future direction. Phytother Res. 2023;37(7):3097–120. 37246823 10.1002/ptr.7861

[216] Sirotkin AV, Harrath AH. Phytoestrogens and their effects. Eur J Pharmacol. 2014;741:230–6. 25160742 10.1016/j.ejphar.2014.07.057

[217] Yuan P, et al. Kaempferol alleviates calcium oxalate crystal-induced renal injury and crystal deposition via regulation of the AR/NOX2 signaling pathway. Phytomedicine. 2021;86:153555. 33852977 10.1016/j.phymed.2021.153555

[218] Rowlands DJ, et al. Equol-stimulated mitochondrial reactive oxygen species activate endothelial nitric oxide synthase and redox signaling in endothelial cells: roles for F-actin and GPR30. Hypertension. 2011;57(4):833–40. 21300668 10.1161/HYPERTENSIONAHA.110.162198 PMC3086276

[219] Bacic D, et al. Involvement of the MAPK-kinase pathway in the PTH-mediated regulation of the proximal tubule type IIa Na+/Pi cotransporter in mouse kidney. Pflugers Arch. 2003;446(1):52–60. 12690463 10.1007/s00424-002-0969-8

[220] Peerapen P, Boonmark W, Thongboonkerd V. Trigonelline prevents kidney stone formation processes by inhibiting calcium oxalate crystallization, growth and crystal-cell adhesion, and downregulating crystal receptors. Biomed Pharmacother. 2022;149:112876. 35367760 10.1016/j.biopha.2022.112876

